# The global significance of biodiversity science in China: an overview

**DOI:** 10.1093/nsr/nwab032

**Published:** 2021-02-23

**Authors:** Xiangcheng Mi, Gang Feng, Yibo Hu, Jian Zhang, Lei Chen, Richard T Corlett, Alice C Hughes, Stuart Pimm, Bernhard Schmid, Suhua Shi, Jens-Christian Svenning, Keping Ma

**Affiliations:** State Key Laboratory of Vegetation and Environmental Change, Institute of Botany, Chinese Academy of Sciences, Beijing 100093, China; Ministry of Education Key Laboratory of Ecology and Resource Use of the Mongolian Plateau and Inner Mongolia Key Laboratory of Grassland Ecology, School of Ecology and Environment, Inner Mongolia University, Hohhot 010021, China; Key Laboratory of Animal Ecology and Conservation Biology, Institute of Zoology, Chinese Academy of Sciences, Beijing 100101, China; Zhejiang Tiantong Forest Ecosystem National Observation and Research Station, School of Ecological and Environmental Sciences, East China Normal University, Shanghai 200241, China; State Key Laboratory of Vegetation and Environmental Change, Institute of Botany, Chinese Academy of Sciences, Beijing 100093, China; Center for Integrative Conservation, Xishuangbanna Tropical Botanical Garden, Chinese Academy of Sciences, Menglun 666303, China; Center of Conservation Biology, Core Botanical Gardens, Chinese Academy of Sciences, Menglun, 666303, China; Center for Integrative Conservation, Xishuangbanna Tropical Botanical Garden, Chinese Academy of Sciences, Menglun 666303, China; Center of Conservation Biology, Core Botanical Gardens, Chinese Academy of Sciences, Menglun, 666303, China; Nicholas School of the Environment, Duke University, Durham, NC 27708, USA; Department of Geography, Remote Sensing Laboratories, University of Zurich, Zurich 8057, Switzerland; State Key Laboratory of Biocontrol, Guangdong Key Laboratory of Plant Resources, Key Laboratory of Biodiversity Dynamics and Conservation of Guangdong Higher Education Institutes, School of Life Sciences, Sun Yat-Sen University, Guangzhou 510275, China; Center for Biodiversity Dynamics in a Changing World (BIOCHANGE) and Section for Ecoinformatics and Biodiversity, Department of Biology, Aarhus University, DK-8000 Aarhus C, Denmark; State Key Laboratory of Vegetation and Environmental Change, Institute of Botany, Chinese Academy of Sciences, Beijing 100093, China; University of Chinese Academy of Sciences, Beijing 100049, China

**Keywords:** biodiversity inventory, biodiversity maintenance, biodiversity monitoring, biodiversity origins, biodiversity-ecosystem functioning

## Abstract

Biodiversity science in China has seen rapid growth over recent decades, ranging from baseline biodiversity studies to understanding the processes behind evolution across dynamic regions such as the Qinghai-Tibetan Plateau. We review research, including species catalogues; biodiversity monitoring; the origins, distributions, maintenance and threats to biodiversity; biodiversity-related ecosystem function and services; and species and ecosystems’ responses to global change. Next, we identify priority topics and offer suggestions and priorities for future biodiversity research in China. These priorities include (i) the ecology and biogeography of the Qinghai-Tibetan Plateau and surrounding mountains, and that of subtropical and tropical forests across China; (ii) marine and inland aquatic biodiversity; and (iii) effective conservation and management to identify and maintain synergies between biodiversity and socio-economic development to fulfil China's vision for becoming an ecological civilization. In addition, we propose three future strategies: (i) translate advanced biodiversity science into practice for biodiversity conservation; (ii) strengthen capacity building and application of advanced technologies, including high-throughput sequencing, genomics and remote sensing; and (iii) strengthen and expand international collaborations. Based on the recent rapid progress of biodiversity research, China is well positioned to become a global leader in biodiversity research in the near future.

## INTRODUCTION

The most striking feature of life on Earth is its incredible diversity. On the one hand, this biodiversity's mere existence begs many fundamental scientific questions: how many species are there on the planet? How and why does biodiversity vary dramatically over the surface of the Earth? Within an ecosystem, how do different species evolve and coexist with each other and their abiotic environments? How does this coexistence change over time, and how does this influence species’ ability to evolve? These questions remain challenging despite recent advances in research [1,2]. On the other hand, biodiversity's functions and services are the basis for sustainable development and human well-being. These services range from primary production, nutrient cycling, purification of water and air, and mitigation of greenhouse gas emissions and climate change, to crop pollination, food and genetic resource provisioning, disease control, and educational, cultural and spiritual benefits [3]. Alarmingly, human-caused global changes in climate and land use are increasingly threatening biodiversity from the poles to the tropics and on land and in the ocean [4]. How is biodiversity responding and adapting to the dramatic changes in the Anthropocene? How can scientists accurately evaluate and predict the risks of local diversity loss and global extinctions and so guide the effective protection of biodiversity? These are the most urgent challenges in biodiversity science [5,6]—the multidisciplinary study of biodiversity in order to conserve it while considering evolutionary and ecological processes, anthropogenic influences and environmental changes affecting it.

China harbours over 35 000 higher plant species [7], over 2700 terrestrial vertebrate species [8] and over 28 000 marine species [9], with a high proportion of endemic species [10]. Among the 36 global biodiversity hotspots (https://www.conservation.org/priorities/biodiversity-hotspots), the ‘Mountains of Southwest China’ hotspot lies almost entirely (>98%) within China, and the ‘Himalaya’ (33%), ‘Mountains of Central Asia’ (29%) and ‘Indo-Burma’ (15%) hotspots lie partly within China. In general, biodiversity in China is much higher than in any other country covering similar latitudes. Three factors may explain this high biodiversity. First, China is among the few countries with the whole spectrum of climate zones, from tropical to cold temperate. Around 40% of the land surface of China rises above 2000 m in elevation. The high geodiversity in China, in turn, creates a wide range of habitats for diverse species. Its mountainous areas serve as macroclimatic and microclimatic refugia for many endemics [11]. Second, the orogeny of the Himalaya, the uplift of the Qinghai-Tibetan Plateau, and the subsequent development of the East Asian monsoon had huge impacts on China's landforms, topography and climate [12]. The result was an elevated rate of species diversification and speciation [13]. Third, during the ice ages of the Pleistocene, large parts of China remained relatively warm and humid, serving as climatically stable refugia for many relict taxa, such as ginkgo (*Ginkgo biloba*) and dawn redwood (*Metasequoia glyptostroboides*) [14]. Besides natural diversity-promoting processes, human activities have reshaped China's biodiversity. Agricultural activities in China date back at least 8000– 9000 years [15], and human-induced loss of natural habitat has caused species decline and losses across several millennia. This process intensified in the 20th century. Rapid increases in human population and urbanization during the last 50 years have pushed a large number of species to the brink of extinction or to extinction in the wild, e.g. Yangtze River dolphins or baiji (*Lipotes vexillifer*), *Rhododendron kanehirae*, and Saiga antelope (*Saiga tatarica*) [16]. Thus, the combined effects of natural and anthropogenic factors have been, and still are, shaping biodiversity patterns in China.

**Figure 1.**
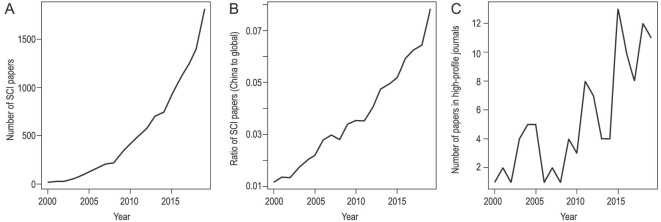
(A) The number of papers on China's biodiversity by China's scholars in each year between 2000 and 2019 published in science citation index (SCI) journals (searched on Web of Science using ‘biodiversity’ and ‘China’ as the topic and ‘China’ as address); (B) its ratio to the number of papers on biodiversity by global scholars (‘biodiversity’ as the topic); and (C) the number of papers on China's biodiversity by China's scholars published in high-profile journals (*Nature, Science, PNAS* and *Nature Communications*).

Over the past two decades, research and publications on China's biodiversity have increased dramatically in quantity and quality. The number of papers published in international journals has grown from a few dozen in 2000 to over 1700 in 2019 (Fig. 1). The Nature Index 2020 Annual Tables highlighted the Chinese Academy of Sciences and several other institutions from China as among the most prolific producers of high-quality research in the natural sciences. Chinese institutions make up all top-ten fastest-rising institutions worldwide, including those in environmental and life sciences (http://www.nature.com/collections/chdeajdica). Both of these are closely related to biodiversity science and conservation. The List of Highly Cited Researchers 2019 (https://clarivate.com/) also shows a good performance from China. Chinese mainland increased its share of Highly Cited Researchers significantly, from 483 or 7.9% in 2018 (covering the period 2006–16) to 636 (10.2%) in 2019 (for the period 2008–18). In 2014, 113 Highly Cited Researchers were from Chinese mainland, whereas by 2019, there were 347, nearly a 3-fold increase.

There are multiple reasons behind this accelerating progress, including increasing financial investment in basic research at national and provincial levels and significant improvements in Chinese ecologists’ and biologists’ research capacity through international collaborations. In addition, the recent establishment of multiple excellent ecological research platforms (e.g. Chinese Forest Biodiversity Monitoring Network, the Biodiversity and Ecosystem Functioning Experiment in China (https://bef-china.com/), and the Chinese National Ecosystem Research Network (http://cnern.cern.ac.cn/en/)) have enhanced understanding of biodiversity across spatial and temporal scales. Building on these successes, there has been an increase in conservation research capacity (e.g. the Chinese Union of Botanic Gardens, the Germplasm Bank of Wild Species, and the National Park-centred protected area system). Policies within various government programmes (e.g. the Grain for Green Programme, Returning Grazing Land to Grassland Project, and Three-North Shelter Forest Programme) [17] and a key national governance strategy for ecological civilization promotes national conservation efforts. They enable restoration and conservation to complement biodiversity and related research. Notably, the Chinese government has invested US$378.5 billion over the past few decades in 16 massive sustainable development projects [17] and has thereby substantially slowed biodiversity decline [18].

**Figure 2.**
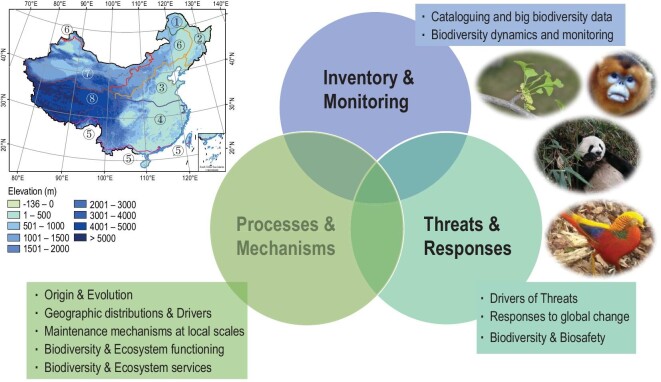
Research highlights of China's biodiversity science in three main areas, including 10 key research topics. The map on the left shows the elevation ranges in China. The lines with different colours in the map divide the country into eight vegetation regions [19]: 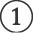 cold-temperate deciduous needle-leaved forests; 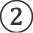 temperate mixed-needle and broad-leaved forests; 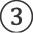 warm-temperate deciduous broad-leaved forests; 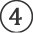 subtropical evergreen broad-leaved forests; 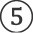 tropical monsoon rain forests and rain forests; 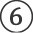 temperate grassland; 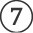 temperate desert; 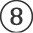 Qinghai-Tibetan Plateau alpine vegetation. Digital elevational model (DEM) data at 10 arcmin spatial resolution were downloaded from WorldClim [20]. The four pictures present the iconic species *Ginkgo biloba* (copyright Yunpeng Zhao), *Rhinopithecus roxellanae* (copyright Sheng Li), *Ailuropoda melanoleuca* (copyright Yibo Hu) and *Chrysolophus pictus* (copyright Sheng Li). The map of China is from http://bzdt.ch.mnr.gov.cn/.

Considering the global significance of China's biodiversity and the marked improvement in biodiversity research by Chinese research institutes over the past two decades, this paper provides an overview of the achievements. Our purpose is to summarize recent advances in biodiversity science by scientists from China and to identify potential barriers and future directions for further advancing biodiversity science, both in China and worldwide. The rich experiences and lessons learned over recent decades also provide useful examples for other countries and regions hoping to follow a similar development path. In reviewing publications with Chinese institutions as the first affiliation in high-profile journals such as *Science* and *Nature* (and other journals in their families) or high-impact systematic efforts, we highlight the significant progress of biodiversity science in China in three critical research areas: (i) inventory and monitoring (what and how many species are present in China, and where are they located? How and why do they change over time?); (ii) mechanisms and processes (origination, evolution and adaptation, environmental relations, and biotic interactions within China's biota); and (iii) threats and responses (how is biodiversity threatened, and how does it respond to global change?). We divide these three areas into 10 key research topics (Fig. 2).

## BIODIVERSITY INVENTORY AND MONITORING

### Species catalogue and big biodiversity data

#### Species catalogue in China

China's rich biodiversity poses a challenge for adequately collating, analysing and managing the enormous amount of biodiversity information, effectively communicating the information to the public, and facilitating the development of biodiversity-related policies. Several generations of Chinese scientists have made significant progress in cataloguing China's species and aggregating big biodiversity data as a fundamental building block for further research to meet these challenges [21].

Researchers at the Chinese Academy of Sciences organized 850 universities and institutes to conduct more than 40 national-scale surveys of natural resources, including biodiversity. They were national-scale efforts in the 1950s–60s and regional-scale surveys in the 1970s–80s. These surveys are responsible for generating much of our knowledge of China's species. For example, the first Qinghai-Tibet Plateau scientific survey during 1973–76 found seven new plant genera, 300 new plant species, 20 new insect genera and 400 new insect species [22]. These large-scale biodiversity investigations provide a solid basis for further species descriptions in China. As the world's largest completed flora, *Flora Reipublicae Popularis Sinicae* (FRPS) started in 1958 and was completed in 2004. It documented 31 141 vascular plant species in 126 books of 80 volumes (Table S1, Fig. S1) [23]. Subsequently, FRPS was revised into the *Flora of China* with 49 volumes in English [24]. *Plants of China: A Companion to Flora of China* followed to answer the question of why there are so many species in China and how the Chinese people have traditionally used them [21]. Recently, both *Fauna Sinica*, with 162 volumes (Table S2), and *Florarum Cryptogamarum Sinicarum*, with 96 volumes, have been published (Table S3). The compilations of floras of most provinces and dozens of provincial-level faunas have been completed, providing detailed descriptions of regional biodiversity. Moreover, the *Fossil Flora of China* (four volumes) summarizes the distributions, research history and characteristics of China's 2248 fossil plant species [25]. The *Catalogue of Exotic and Cultivated Plants in China* appeared in 2018 and documents 13 635 exotic plant species and 13 941 native cultivated plant species [26] (Fig. S1). Based on the *Checklist of Marine Biota of China Seas* [27] and other information, *The Living Species in China's Seas* documented more than 28 000 marine species belonging to 59 phyla in China's seas [9].

**Figure 3.**
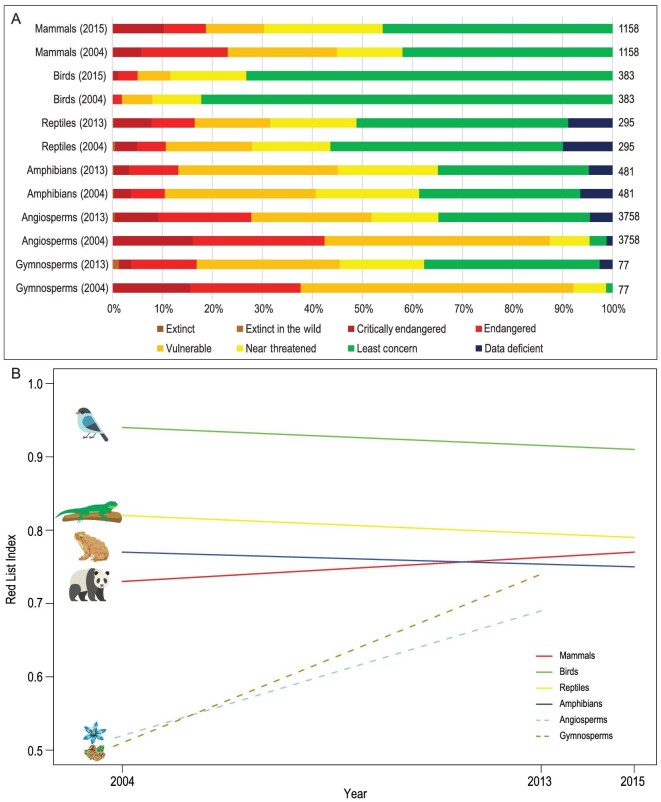
Trends in IUCN listings of threatened species in China. (A) Changes in the threatened ranks of gymnosperms, angiosperms, amphibians, reptiles, birds and mammals in China. The numbers at the end of bars were the numbers of species assessed. (B) Red List Index for gymnosperms, angiosperms, amphibians, reptiles, birds and mammals in China. The first assessment was in 2004, and the second in 2013 for plant species and 2015 for animal species. Red List Index is an indicator of overall extinction risk for a set of species. It is used to track meaningful trends in biodiversity status simply by looking at overall changes in threat status of the same set of species between updates [31].

The first version of *Catalogue of Life: China* (CoL-China) was released in 2008, and it has been updated annually. The 2020 version includes 122 280 species and intraspecific taxa covering different taxonomic groups (http://www.sp2000.org.cn). The CoL-China also provides a real-time update for new species to fill the existing gaps in the inventory, particularly for terrestrial arthropods and fungi. For effective conservation and management of biodiversity, Chinese researchers adopted the International Union for Conservation of Nature (IUCN) Red List categories and criteria to evaluate species threat status. There were 4408 seed plant species and 3351 vertebrate species in the first assessment in 2004 [28], and 34 450 higher plant species and 4357 vertebrate species in the second assessments in 2013 and 2015, respectively (Table 1) [29]. The threat status of 9302 out of >10 000 species of macrofungi was also assessed in 2016 [30]. Trends in overall threat status for groups of species are indicated by changes of species numbers in threatened status of successive Red List assessments (Fig. 3) [31]. Based on the two successive species Red List assessments, indicators showed that the conservation statuses of birds, reptiles and amphibians are declining, while mammals, gymnosperms and angiosperms are improving (Fig. 3). These improvements suggest that current conservation activities are relatively effective for most mammals, gymnosperms and angiosperms. In contrast, urgent actions, which are different from current conservation approaches, are needed to reverse the decline of birds, reptiles and amphibians.

**Table 1.** Number of species, endemic species and threatened species for well-studied taxonomic groups in China.

Taxonomic group	Number of species^a^	Number of endemic species	Number of threatened species
**Bryophytes**	3021	524	186
**Ferns**	2129	842	182
**Gymnosperms**	237	88	148
**Angiosperms**	28 996	14 693	3363
**Amphibians**	408	272	176
**Reptiles**	461	142	137
**Birds**	1372	77	146
**Mammals**	673	150	178

^a^The number of species and endemic species is from Gao *et al.* (2018) [10], and the number of threatened species is from Zang *et al.* (2016) [29].

Despite recent progress, many taxonomic groups, such as invertebrates and fungi, have not been thoroughly inventoried. We still lack sufficient data to assess the threat to them. Second, some remote areas such as south-eastern Tibet and border areas in Yunnan province have not been well inventoried for most taxonomic groups. Finally, only 10% of specimens in Chinese collections have precise geographic localities. Moreover, some species are certainly misidentified [32]. These gaps in our knowledge may bias further biogeographic analysis and biodiversity conservation planning. Further research should explore poorly inventoried taxonomic groups and areas and check questionable identifications of herbarium specimens using DNA barcoding. Such research may elucidate mechanisms of biodiversity formation at biogeographic scales by integrating the big data from species catalogues with data about the tree of life, geological history and other factors influencing species distributions over time.

#### National Specimen Information Infrastructure and big biodiversity data

In recent decades, increasing effort and financial investment have enabled the development of many biodiversity databasing platforms. Dozens are now online [33]. The Chinese National Specimen Information Infrastructure (NSII; http://www.nsii.org.cn/) was established in 2003 to document historical and current distributions of species in China. NSII also includes multiple subplatforms, such as the Chinese Virtual Herbarium (http://www.cvh.ac.cn/), National Animal Collection Resource Center (http://museum.ioz.ac.cn/), Specimen Resources Sharing Platform for Education (http://mnh.scu.edu.cn/), Digital Specimen Sharing Platform for Nature Reserves (http://www.papc.cn/), Chinese Field Herbarium (https://www.cfh.ac.cn/) and Plant Photo Bank of China (http://www.plantphoto.cn/). NSII has already digitized 15.7 million specimens from 329 Chinese herbaria and museums and 13 million colour photographs and other items (http://www.nsii.org.cn/). These species catalogues and digitized specimens provide essential information on the names, taxonomic relationships and distributions of life in China and play critical roles in understanding species origins, evolution and biodiversity conservation [13,34,35].

In 2018, CAS initiated the Big Earth Data Science Engineering Project, with a budget of US$250 million over five years (CASEarth, http://www. casearth.com). One of the critical elements of CASEarth is to integrate available biodiversity information for users from the academic community, decision-makers, conservation practitioners and the general public (http://www.bio-one.org.cn/). Citizen science is an increasingly important mechanism for collecting data on species distributions across space and time. Citizen science approaches for bird species have been well developed and have been used to identify almost nationwide potential habitats of 1111 bird species, including 167 nationally protected species and 70 threatened species. Nearly 25% of the nationally protected species and 20% of the threatened species use farmland as habitat [36]. Thus, new agricultural systems in China should develop to integrate traditional and scientific knowledge of sustainable intensification towards conserving focal species in high-priority areas.

#### Vegetation maps and ‘vegegraphy’ of China

With the efforts of three generations, including over 200 Chinese vegetation scientists, the *Vegetation Atlas of China* (1 : 1 000 000) and a vegetation division map (1 : 10 000 000) were both published in 2001, with 11 groups of vegetation types and 796 formations [37]. The two maps were subsequently updated with 960 formations and subformations and 116 vegetation regions [38]. Since the two vegetation maps were based on vegetation surveys in the 1980s, China's vegetation map was recently updated with 12 vegetation types and 866 vegetation formations/subformations. About 3.3 million km^2^ of China's vegetated area has changed vegetation type since the first vegetation map in the 1980s [39]. The *Vegetation of China* summarized the floristic composition and distribution of the main vegetation types [40]. Chinese vegetation scientists have started to write monographs about particular vegetation units defined in their system of vegetation classification. They introduced the new term ‘vegegraphy’ for this type of monograph. The first volume of *Vegegraphy of China* (about spruce forests) was published in 2017 [41].

### Biodiversity dynamics and monitoring

Many regions in China simultaneously display a mix of positive and negative biodiversity trends. Several national-scale biodiversity monitoring networks have been established across major ecosystems and nationwide environmental gradients to enable monitoring. These networks include the Chinese Biodiversity Observation and Research Network (Sino-BON), China Biodiversity Observation Network (China-BON), Chinese National Ecosystem Research Network (CNERN), Chinese Ecosystem Research Network (CERN), Chinese Forest Ecosystem Research Network (CFERN) and National Forest Inventory. These networks have statistically rigorous designs with conceptual models of biodiversity to quantify and understand these trends and their drivers. They provide forewarning of biodiversity loss and enable adaptive management of biodiversity for policymakers [42].

#### Sino-BON and China-BON

**Figure 4.**
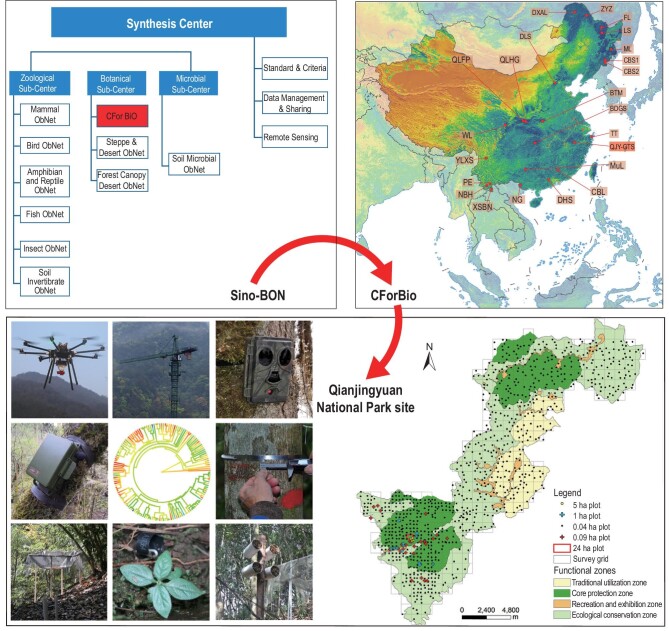
An illustration of how Sino-BON is organized from sites (Qianjiangyuan National Park, GTS) to subnetwork (CForBio) and national-scale network (Sino-BON) for monitoring dynamics of species and ecosystems, and multiple trophic interactions through cooperation among the 10 subnetworks. Top left panel: structure of Sino-BON (Chinese Biodiversity Monitoring and Research Network) including a synthesis centre and zoological, botanical and microbial sub-centres. The Zoological sub-centre includes six subnetworks (ObNet): Mammal, Bird, Amphibian and Reptile, Fish, Insect and Soil Invertebrate. The Botanical sub-centre includes three subnetworks: CForBio (Chinese Forest Biodiversity Monitoring Network), Steppe and Desert, and Forest Canopy. The microbial sub-centre includes only the microbial subnetwork. Top right panel: 20 large forest dynamics plots along latitude in Eastern China. Bottom right panel: 1 km × 1 km grids in four zones of Qianjiangyuan National Park. Bottom left panel: biodiversity monitoring at the Park from top to bottom: drone for near-surface remote sensing, forest crane for canopy biodiversity, infrared-triggered camera for mammals and birds, automated acoustic recording device for songbirds, phylogeny of woody species with molecular data, dendrometer measuring the growth of trees, seed rain trap, seedling plot, insect nest trap. The map of China is from http://bzdt.ch.mnr.gov.cn/.

Sino-BON and China-BON monitor biodiversity change in multiple taxonomic groups, including plants, animals and microbes. The Chinese Academy of Sciences initiated Sino-BON in 2013. It is composed of 10 subnetworks, including three subnetworks of plant diversity (CForBio, Steppe & Desert Network and Forest Canopy Network), six subnetworks of animal diversity (Mammal, Bird, Amphibian and Reptile, Insect, Soil invertebrate, and Freshwater fish), and a subnetwork of soil microbial diversity. For each subnetwork, several sites have been established across China [43] (Fig. 4). The Ministry of Ecology and Environment of China initiated China-BON in 2011. It now has four subnetworks (Mammals, Birds, Amphibians and Butterflies). China-BON now includes more than 440 observation sites and 9000 line or point transects to cover representative ecosystems in China [44].

Sino-BON has established a multidisciplinary research platform to monitor species and ecosystems’ dynamics and multiple trophic interactions through cooperation among the 10 subnetworks, as illustrated in Fig. 4. CForBio has established 23 large forest dynamics plots in one subnetwork, each with an area around 20 ha, covering forests from boreal to tropical zones. The plots monitor 1893 woody species and more than 2.69 million tree individuals with a total plot area of 658.6 ha [45]. In some of the CForBio plots, the Sino-BON-Forest-Canopy subnetwork has equipped eight forest cranes to monitor forest canopy microclimate and its diversity of plants, arthropods and microbes [46]. The Sino-BON-Mammals subnetwork has set up 20–150 infrared-triggered cameras in 1 × 1 km grids of 30 representative natural forests to monitor terrestrial mammals and ground-dwelling birds. The Sino-BON-Birds subnetwork has established 16 international and 38 domestic sites across the Eurasian continent to monitor 2569 migratory individuals of 63 bird species using remote telemetry devices [43].

#### CERN, CFERN and CNERN

To observe long-term changes in ecosystem functions, the Chinese Academy of Sciences initiated CERN in 1988. It now consists of 44 field stations covering all major ecosystems in China (http://www.cern.ac.cn). Using a standardized monitoring and data quality-control system, most CERN stations have accumulated data over 30 years. These provide unparalleled insights into ecosystem dynamics and changes. An example is the long-term observational data on soil organic carbon in the top 20-cm layer in old-growth forests in southern China from 1973–2003. There was an accumulation of atmospheric carbon at an unexpectedly high rate. It highlighted the importance of establishing long-term observational studies on the responses of belowground processes to global change [47]. The former Ministry of Forestry of China initiated CFERN in 1992 and currently has 110 field stations covering nine forest types (https://cfern.org/). It includes two main forest transects: the North–South Transect of Eastern China, along temperature and precipitation gradients in Eastern China, and the West-East Transect of Southern China, along the Yangtze River from near sea level to altitudes higher than 6000 m on the Qinghai-Tibetan Plateau [48]. To integrate, standardize and coordinate the above-described platforms sponsored by different sectors in China, the Ministry of Science and Technology of China sponsored CNERN. It seeks to integrate observation facilities and data resources and standardize research methods, tools and protocols. Currently, CNERN has 53 field stations covering China's main ecosystems, selected from well-equipped and developed stations in CERN, CFERN and others (http://cnern.cern.ac.cn/en/).

#### National Forest Inventory

The State Forestry and Grassland Administration of China has established a national-scale forest inventory system to monitor forest resources and biodiversity. The national forest inventory has been conducted at five-year intervals since 1973. The ninth inventory was in 2018 (http://www.forestry.gov.cn/gjslzyqc.html). A systematic sampling design uses 2 × 2 km to 8 × 8 km grids in all provinces to conduct a qualitative assessment. Permanent plots (0.0667 ha each) have standardized measurements in each plot, including tree diameter at breast height (DBH) and height of trees with ≥5 cm DBH. Since the seventh inventory (2004–8), 415 000 sampling plots were surveyed for each inventory. From the first to the ninth inventory, the forest coverage in China increased from 12.69% to 22.96%, and the total growing stock volume from 8.66 to 17.56 billion m^3^ [49]. This platform monitors forest resources dynamics in China and accounts ecosystem services, such as carbon stocks, and their interactions with biodiversity and human disturbance [50].

## PROCESSES AND MECHANISMS

### Origin and evolution of China's biodiversity

#### Uplift of the Qinghai-Tibetan Plateau created a cradle for biodiversity evolution

As an evolutionary cradle of biodiversity, many lineages in China originated with topographic changes and climate shifts, such as the uplift of the Qinghai-Tibetan Plateau and surrounding regions, the development of the East Asian monsoon, and the aridification of Western China [51]. There is increasing evidence that a proto-Tibetan Plateau had probably already developed in Cretaceous-Paleogene times (∼60 Ma; ‘Ma’ meaning ‘million years ago’) before the collision of India with Asia. A generalized expansion of the Qinghai-Tibetan Plateau started no later than the early Miocene to Pliocene (25–5 Ma). It was accompanied by continued surface uplift of high mountain ranges and the rise of a wide east–west orientated deep central valley until the Neogene [52,53]. Chinese biologists have provided striking examples of how these geographical developments continuously shaped lineage diversification for various taxonomic groups. For example, a series of geological events such as the growing Eurasian land from the Tethyan region (about 40 Ma), the collision of the Indian and Asian plates during the Eocene (37–34 Ma) and the closure of the Turgai Strait (∼29 Ma) [54] drove the adaptive radiations of Holarctic amphipods of the genus *Gammarus*.

Similarly, Hynobiidae, an ancient lineage of living salamanders, diversified with the continuous uplift of the Qinghai-Tibetan Plateau and its neighbouring regions [55]. The concept of a ‘proto-Tibetan Plateau’ with a flat centre is contentious. For examples, well-preserved fossil palm leaves suggest an extensive valley system with a floor lower than 2.3 km above sea level in central Tibet during the late Paleogene (25 Ma) [56]. Moreover, the south-eastern margin of Tibet in the latest Eocene (∼34 Ma) was likely to have been ∼3000 m above sea level and attained its present elevation only in the early Oligocene [57].

After India's collision with Asia, a burst of terrestrial faunal and floral diversification occurred in China and neighbouring areas. For example, both mountain building and intensification of the Asian monsoon likely accelerated diversification and colonization of Asian frogs of the tribe Paini (Anura: Dicroglossidae) in freshwater ecosystems [58]. Temperate alpine flora emerged from early Oligocene and its diversification peaked in the middle Miocene in Tibet-Himalaya-Hengduan regions [59,60]. Some high-alpine vertebrate species acquired anatomical or physiological adaptations to extreme environments [61–64], while modern alpine plant genera started to diversify on the Qinghai-Tibetan Plateau during the Late Miocene [65].

After the Miocene, along with global cooling in the Pliocene and recent glacial cycles, a new period of diversification started on the Qinghai-Tibetan Plateau and its margins. For example, a Pliocene mammal assemblage was found from a high-altitude basin in the western Himalayas, with cold adaptations such as the woolly rhinos (i.e. *Coelodonta tologoijensis* and *C. thibetana*) [66]. These findings show that cold-adapted mammals started to diversify within this region in the Pliocene [66,67].

Recent studies support species diversification through multiple mechanisms such as allopatric speciation, pollinator-mediated isolation and diploid hybridization on the Qinghai-Tibetan Plateau [68]. Recently, a novel model of speciation may underpin the formation of the region's high biodiversity, known as the ‘Mixing-Isolation-Mixing’ (MIM) model. Unlike the classic allopatric model with full geographical isolation between populations, the MIM model allows gene flow to shuffle adaptive gene complexes built up during isolation to generate many new combinations and thus diversify exponentially. By combining analyses of genomic data and historical geographic information, mangrove speciation appears driven by multiple MIM cycles of opening or closing of sea straits during historical sea level fluctuations [68]. Similarly, recurrent cycles of admixture and isolation between heterogeneous habitats in sky-islands have driven intraspecific population diversification of the cushion willow (*Salix brachista*) and likely many other species in the Hengduan Mountains [69].

#### Refugia acting as an evolutionary museum for relict species

China is also an evolutionary museum. Qiu *et al.* (2011) synthesized previous phylogeographic studies to identify refugia of relict species, including those near the south-eastern edge of the Qinghai-Tibetan Plateau (Hengduan Mountains, Yungui Plateau), the Three Gorges Mountains Region, and many other mountain ranges such as the Qinling and Nanling ranges [70]. In addition to these refugia, Zhao *et al.* (2019) also recently identified three refugia for ginkgo in south-western, southern and eastern China [71]. Tang *et al.* (2018) identified long-term climatically stable refugia for relict plant species in south-western China and northern Vietnam [14].

At a national scale, China is both a museum and a cradle of biodiversity, while it also has multiple biogeographic origins from other continents. The analysis of a dated phylogeny of Chinese angiosperms and occurrence data revealed that eastern China (humid to semi-humid areas) has served as a floristic museum, by a signature of older divergence in its whole flora, as well as both a museum and a cradle for woody genera [13]. In contrast, western China (arid to semi-arid areas) is an evolutionary cradle for herbaceous genera, shaped by recent diversification in its flora [13]. Contrastingly, 38% of plant taxa in Eastern Asia arrived from other floras in the Northern Hemisphere and the Gondwana. Nonetheless, about 48% of plant taxa in Eastern Asia have an *in situ* origin, providing additional evidence of China's role as a floristic cradle [72].

#### Domestication of plants and animals

The domestication history and rapid evolution of domestic plants and animals have puzzled evolutionary biologists since Darwin. At least 20% of the world's cultivated species (136 out of 666 species) originated from China, where more than 8000 years of selection shaped modern varieties of Chinese crops and livestock [21]. These examples provide an excellent opportunity for the study of domestication [15]. Modern techniques allow reconstruction of the history of plant and animal domestication. This history highlights the role China has played in the domestication of global staple crops and livestock. As examples, dogs were domesticated from wolves in southern East Asia ∼33 000 years ago [73]. Silkworms (*Bombyx mori*) may have been initially domesticated in China as tri-moulting lines. They spread independently along the Silk Road, which resulted in the development of local varieties [74].

Studies on domestic plants and animals also unravel the mechanisms of rapid evolution behind artificial selection, providing insights into the past. It also suggests strategies for future crop and livestock breeding, considering conditions to ensure their survival under future climates. For example, under artificial selection, some of the candidate genes highly expressed in the Chinese native dogs’ (*Canis lupus familiaris*) brain evolved rapidly. They were partially responsible for dogs’ behavioural transformation [75]. Comparative genomics revealed the role of interspecific gene flow in cattle (*Bos taurus*) domestication. For example, Tibetan cattle obtained the gene for hypoxia adaptation from sympatric yaks (*Bos grunniens*) [76]. Rice (*Oryza sativa*) and wheat (*Tricicum aestivum*) are major global crops. Through selection and breeding, crops have undergone significant phenotypic changes in flowering time, grain size, colour, shattering, seed dormancy and tillering. For example, a minor-effect quantitative trait locus, DTH2 (days to heading protein on chromosome 2), has been shown to probably represent a target of human selection for adaptation to long day length [77]. Likewise, RNA-seq techniques have revealed the mechanisms for the enhanced environmental adaptability and improved grain quality of hexaploid wheat (*Tricicum aestivum*, AABBDD) compared with its diploid progenitor (*Aegilops tauschii*, DD) and tetraploid progenitor (*Triticum turgidum*, AABB). Expansion of agronomically relevant gene families in *Aegilops tauschii* is associated with disease resistance, abiotic stress tolerance and grain quality [78].

### Geographical distribution of biodiversity

As a consequence of differences in evolutionary history, topography, contemporary and past climate, species richness and diversity patterns vary across China. Many studies have tested the effects of these different temporal-spatial factors on Chinese biodiversity from different perspectives, including taxonomic, phylogenetic and functional diversity [34,79,80]. Our understanding of these various drivers’ contribution to biodiversity patterns across China is growing, providing insights into mechanisms and drivers behind diversity patterns elsewhere.

#### Taxonomic diversity distribution and the underlying drivers

Species distributions and species richness patterns, and their multiple-scale drivers have been widely studied in China. Specifically, regions with relatively stable glacial-interglacial climate (most notably south-western China) have higher proportions of both endemic bird species and endemic plant species [81,82]. Contemporary temperature is positively associated with overall tree species richness in China and North America, perhaps supporting the metabolic theory of ecology [34]. Notably, Chinese-led research has also shown that glacial-interglacial climate change, along with contemporary climate, topographic heterogeneity, and species traits, determine the range-size of terrestrial vertebrates at the global scale [83].

In addition to these natural drivers, to reflect China's long cultural history and dense human population, many studies have addressed the consequences of anthropogenic activities across past decades, centuries and millennia in China. Those activities have impacted the range dynamics of mammals and plants and the distribution and population size of threatened species [84–88]. For example, human disturbance constrains herbivorous waterfowls’ ability to track the ‘green wave’ of food availability along their spring migration routes [89]. Extensive artificial shorelines, loss of essential intertidal and wetland regions, global warming, and larval transport are the main factors limiting distributions of intertidal invertebrates along the Chinese coast [90]. Notably, a large-scale field experiment conducted in subtropical regions in China and subarctic regions in Norway found that nutrient enrichment can modify temperature-biodiversity relationships in aquatic microcosm communities [91].

#### Phylogenetic and functional diversity distribution and the underlying drivers

While taxonomic diversity treats species as ecologically equivalent, phylogenetic diversity and functional diversity better reflect species’ evolutionary and ecological differences [79,80]. Besides higher plant species richness, China also has higher plant phylogenetic diversity than Europe and North America, reflecting their different histories [92,93]. Phylogenetic diversity for both gymnosperms and angiosperms in China also declined with increasing contemporary climatic stress, consistent with a role for tropical niche conservatism in shaping China's plant diversity [80]. More frequent phylogenetic clustering than overdispersion in Chinese terrestrial vertebrates suggests essential roles of both regional ecological and evolutionary factors, such as environmental filtering and intra-regional speciation, in shaping animal assemblages [94].

As an important facet of biodiversity, functional diversity links to ecosystem function and ecosystem services across temporal-spatial scales [79,95]. Notably, an influential global study found that root trait diversity was greatest in the tropics. It declined sharply in temperate and desert biomes due to both evolutionary history and soil resource supply [79]. Another global study showed that water availability influenced the relationship between maximum plant height and hydraulic traits, e.g. taller species from wet regions had greater xylem efficiency and lower hydraulic safety [96]. At the national scale, precipitation, plant life-form and evolutionary history together affect the relationship between the type of leaf margins of Chinese woody plants and temperature [95]. A recent national study also found that contemporary climate affects the geographical variation in sexual systems of Chinese woody plants indirectly through its effects on mature plant height [97].

### Biodiversity maintenance in local communities

How species coexist in diverse communities where many species share similar resource requirements has been an enduring puzzle for community ecologists. Classical theory shows that stable coexistence occurs when stabilizing niche differences outweigh fitness differences by causing species to more strongly limit themselves than limit their heterospecific competitors [98]. The processes that determine the distribution and abundance of species include dispersal limitation, abiotic filtering, resource competition and trophic interactions with natural enemies and mutualists. In the context of rapid degradation and destruction of natural ecosystems, there is an urgent need to understand the underlying mechanisms of species coexistence that may protect species from local extinction and inform ecosystem restoration. Furthermore, the resilience of a community to environmental changes often stems from a level of redundancy. Understanding the role of species and guilds would facilitate a clearer understanding of systems’ resilience and thereby enable restoration to ensure continued ecosystem function.

#### The prevalence of conspecific density dependence in plant communities and the underlying mechanisms

Conspecific negative density dependence (CNDD) may be a major mechanism for the maintenance of plant diversity. Proximity to conspecific adult plants reduces seedling survival through resource competition and attacks by host-specific pathogens, herbivores or predators [99]. Covering the complete climatic spectrum in China, the analyses of forest dynamics plots have provided strong evidence for CNDD in temperate, subtropical and tropical forests [100]. For instance, density dependence can be a key factor in structuring seedling survival patterns at very local scales. This can, in turn, result in a community showing compensatory trends [101]. In some cases, negative interactions also operate among neighbours of closely related species [102], but this effect's strength is smaller than for conspecific neighbours [100]. By incorporating multiple life stages and numerous influential factors, the strength of CNDD also varies with tree life stages and fluctuating environments, with the strongest CNDD at early life stages [103]. In addition, recent observational work with a tropical species has shown that seedlings near closely related conspecific neighbours have reduced growth performance [104]. Further experimental work suggests that soil microbes’ within-species specialization helps explain such intraspecific variation in CNDD [105].

**Figure 5.**
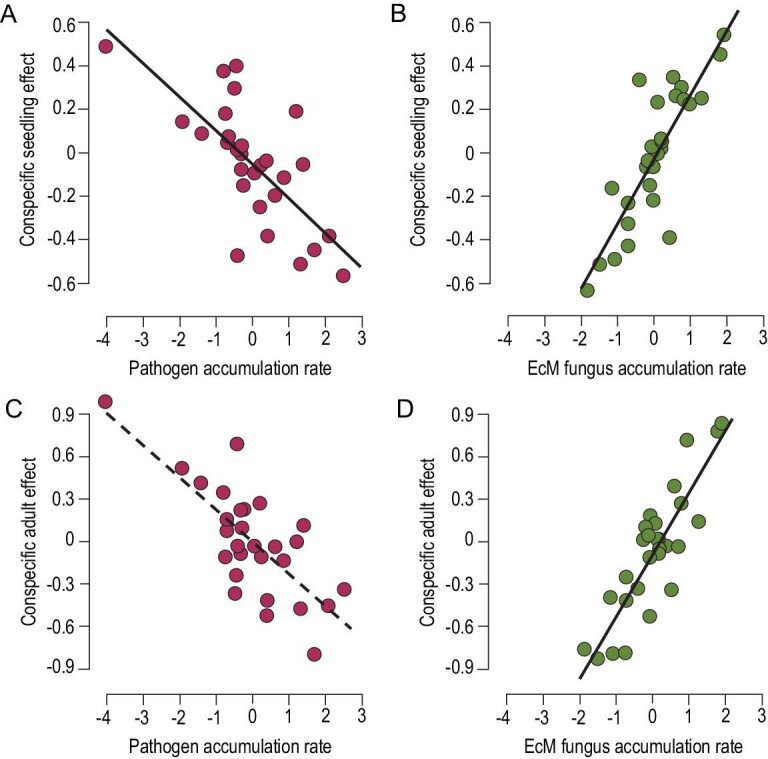
Relationships between species-specific CNDD effect and pathogenic and EcM fungus accumulation rates demonstrating that species coexistence in subtropical forests depends on the interplay between mutualistic and pathogenic fungi [110]. Solid lines and dashed lines indicate significant and non-significant effects at *P* < 0.05, respectively. Each point represents a tree species with different mycorrhizal associations.

Although CNDD has been detected widely in plant communities, empirical support for the prediction that CNDD caused by natural enemies can translate into enhanced community diversity remains limited. Key studies in subtropical forests combined with field surveys and shade-house experiments have revealed that adult trees cause density-dependent mortality in conspecific seedlings by regulating the frequency of pathogenic soil fungi [106]. Such pathogen-mediated density dependence could translate into ‘rare species advantage’ in the community [107]. Moreover, tree species’ adverse effects on seedling survival extend to their close relatives because of phylogenetic conservatism in host-specific interactions between plants and their pathogens [108]. Similar patterns were also evident in temperate forests, where natural enemies, such as plant-associated fungi and insect herbivores, reduce seedling recruitment and survival at high adult conspecific density. This pattern is especially pronounced for ectomycorrhizal and shade-tolerant species [109]. It suggests that plant–pathogen interactions and feedbacks through host-specific changes in soil communities are important for plant species coexistence. More importantly, a recent empirical study provided compelling evidence that both harmful pathogenic fungi and beneficial ectomycorrhizal (EcM) fungi shape interspecific variation in the strength of CNDD (Fig. 5). Pathogenic fungi may play a key role in driving tree interactions’ strength but can be overruled by EcM fungi [110]. Further studies evaluating the possible links between CNDD, pathogens, mycorrhizal symbiosis and local plant species diversity may explain how numerous plant species can coexist in natural communities.

#### Functional traits, phylogeny and community assembly

To understand the processes underlying community assembly, Chinese ecologists have studied the influence of biotic and abiotic factors on plant phylogenetic and functional diversity and composition. Resource partitioning, such as interspecific habitat differences in topography and soil nutrients, and dispersal limitation, has emerged as an important explanatory factor for multiple biodiversity patterns in China's plant communities. The patterns included species–area relationships, variation in community composition across space or time (β diversity) and functional alpha diversity [111,112]. At a global scale, evolutionary history and the co-evolution of key functional traits, such as root and hydraulic traits, have been explored by Chinese scientists to determine functional trade-offs along large-scale environmental gradients [96,113,114]. Chinese ecologists have proposed new research pathways. For example, intraspecific trait variation may predict demographic rates better than species-level traits. The latter cannot capture some dimensions of tree function critical for demographic rates [115]. ‘Community-level traits’ have been proposed to broaden the applicability of functional traits to large-scale patterns and their generating mechanisms [116]. By sequencing the transcriptome of gene ontologies for light use and harvesting among co-occurring species, the functional genomic similarity of these genes has the potential to increase the breadth and depth of our understanding of how gene function influences community assembly [117].

### Biodiversity and ecosystem functioning

The tremendous diversity in China is at risk from rapid economic development, its large human population, and climate change, destroying biodiversity and harming ecosystem functioning [17]. China's high biodiversity and its regional heterogeneity provide an exceptional opportunity to conduct controlled experiments and field surveys across natural gradients to understand how the processes maintaining biodiversity contribute to ecosystem functions. Chinese ecologists have recently strived to understand the mechanisms behind the relationship between biodiversity and ecosystem function in both forest and grassland ecosystems. One element of this research involves biodiversity–ecosystem functioning (BEF) experiments across various ecosystem types. Generally, they found that increasing biodiversity leads to increased ecosystem functions—such as productivity and stability—a relationship that appears across scales. This relationship is likely mediated by the biotic interactions across trophic levels and interspecific trait variation.

#### BEF relationships in forest ecosystems

The largest BEF experiment in forest ecosystems started in 2009/2010 in subtropical China (referred to as BEF-China). Among 26 BEF platforms around the world, BEF-China is the forest experiment with the largest number of species (42 tree species and 18 shrub species), the largest area (around 40 ha) and the highest range of species richness (from monocultures to mixed forests with 24 tree species) (http://www.treedivnet.ugent.be/). The design of the species mixtures considered differences among taxonomic, phylogenetic, functional diversity and genetic diversity [118]. This experiment extrapolated the findings of previous grassland experiments. It showed that biodiversity roughly doubled plant productivity and carbon storage across the tested range of species richness. The positive effects of biodiversity increased significantly over eight years of plant growth [119]. BEF-China also includes plots in a neighbouring national park's natural forests to compare with experimental stands [120]. The current findings suggest that restoring biodiversity based on multispecies afforestation strategies can effectively mitigate climate change and provide many other benefits and services.

In subtropical forests, the biotic interactions across trophic levels and interspecific trait variation mediated the mechanisms underpinning the observed positive BEF relationships, rather than intraspecific genetic diversity [121–123]. For example, plant diversity had non-random dilution effects on root pathogenic infection [121], while phylogenetic diversity of herbivore communities decreased with increasing tree phylogenetic diversity [122]. Species richness effects were more related to interspecific trait variation than intraspecific genetic diversity [123].

#### BEF relationships in grassland ecosystems

China has established a series of manipulative BEF experimental platforms in glasslands. They include a BEF experiment with plant removal in Inner Mongolia [124], a BEF platform on the Qinghai-Tibetan Plateau [125] and an experiment studying the effects of livestock diversity on grassland ecosystem functioning in north-eastern China [126]. These manipulative experiments provide the platforms for a deeper understanding of the ecological mechanisms of BEF relationships and the response of plant communities to global change.

The positive biodiversity-stability relationship appears in different grasslands, likely caused by asynchronous dynamics among ecosystems’ components. Using a 24-year study of Inner Mongolian grassland, Bai *et al.* (2004) documented plant diversity's positive influence on ecosystem stability [127]. In species-rich communities, they found that ecosystem stability increased from species-level to functional-group to whole-community level due to asynchronous dynamics among major components at both species and functional-group levels. Similarly, in alpine grassland on the Qinghai-Tibetan Plateau, a long-term climatic warming experiment over 30 years (1983–2014) showed increased grass abundance and decreased sedge abundance. It did not affect the total aboveground net primary productivity [128]. This demonstrated that in species-rich ecosystems shifting species composition in response to climate warming may stabilize primary production by shifting from aboveground to belowground productivity.

In contrast, the BEF experiment in an alpine site on the Qinghai-Tibetan Plateau found that warming, rather than precipitation change, reduced temporal stability over five years. It did so by altering the asynchronous population dynamics of the dominant species [129]. The combined effects of above- and belowground biodiversity explained 45% of the variation in ecosystem multifunctionality [125]. Yet high livestock diversity (sheep and cattle grazing vs. sheep or cattle or no grazing) increased ecosystem multifunctionality by increasing diversity of a range of taxonomic groups, including plants, insects, soil microbes and nematodes. This relationship was much clearer when the analysis correctly accounted for the different components of diversity and the different functions [122].

At a national scale, carbon storage increased with increasing biodiversity, supporting the generality of the relationship. Based on 6098 forest, shrubland and grassland sites across China, soil organic carbon storage was substantially enhanced by increasing species richness and belowground biomass. Species richness, aboveground net primary productivity, and belowground biomass can be as important as environmental drivers’ direct effects such as precipitation and temperature [130].

### Ecosystem services and socio-economic development

Biodiversity enhances many ecosystem services, such as buffering climate extremes, regulating pests and producing food and wood, in addition to cultural identity and aesthetic inspiration [3]. However, recent biodiversity loss due to anthropogenic activities has weakened ecosystem services by altering ecosystem functioning and stability at broad scales [131]. In China, scientists have recently made several comprehensive assessments of ecosystem services at both regional and national levels, including evaluating the complex relations between ecosystem services and socio-economic development. They propose adding the results of ecosystem services assessments into ecological planning to enable integrated assessments and planning.

#### The assessment of ecosystem services

To reduce the risks of natural disasters and restore natural capital, China has invested over US$50 billion through the Natural Forest Conservation Programme and the Sloping Land Conversion Programme between 2000–9. These programmes have enabled the continued provision of various ecosystem services, including food production, carbon sequestration, soil retention, flood mitigation, sandstorm prevention and water retention [132]. Another assessment of China's six key ecological restoration projects suggested that the total annual carbon sink in the regions covered by these projects between 2001–10 was estimated to be 132 Tg carbon per year. Over half of this carbon was from implementing these projects [133]. Notably, the value of ecosystem services provided by the giant panda (*Ailuropoda melanoleuca*) and its reserves is about 10–27 times higher than the cost of supporting these reserves [134]. Ecosystem services provided by other taxa are likely understudied. Examples include pollination services provided by birds, bats and insects.

#### The balance between ecosystem services and socio-economic development

Ecosystem services are the benefits nature provides to people. That said, strategies for improving ecosystem service provision may conflict with local socio-economic development. For example, the revegetation programmes in China's Loess Plateau increased the net primary productivity to an upper limit for the water resources. This limit may threaten the provision of sufficient water for human demands [135]. The Relocation and Settlement Programme of Southern Shaanxi Province would benefit local governments, downstream water consumers and global beneficiaries over the long term. Short-term costs to residents and government may outweigh these benefits, however [136]. In south-west China, strict conservation of rain forest would provide greater values from many ecosystem services than ‘cash forest monocultures’. Nonetheless, local people still prefer to plant monoculture forests, e.g. for rubber and tea [137]. Yet, well-planned initiatives can provide mutual environmental and economic benefits. An example is the Paddy Land-to-Dry Land programme in Beijing. It successfully improved water quantity and quality more than it reduced agricultural output, creating benefits that were more than five times greater than the costs [138]. Exploring synergies provides a mechanism to maximize benefits and enable both the satisfaction of human needs and biodiversity maintenance.

#### Adding ecosystem services into ecological planning

Ecosystem services matter to people, and therefore programmes for landscape planning should incorporate them. For example, in a framework for drawing the ecological redline areas in Shanghai, the inclusion of ecosystem services in planning would substantially increase terrestrial habitat protection and decrease the trade-offs between development and environmental quality [139]. A national assessment of protected areas in China found that China's nature reserves have not played a large enough role in protecting both biodiversity and key ecosystem services [35]. These authors suggest establishing a new protected area category that includes integrated consideration of biodiversity, ecosystem services and human activities. National level redline programmes have also been developed to maintain ecosystem services and biodiversity. Such approaches provided an additional mechanism to maximize ecological security. They also illustrate how environmental policy within China has a solid scientific basis.

## THREATS AND RESPONSES

Species endangerment is often the result of a combination of extrinsic and intrinsic factors, which lead to declines in populations and long-term viability. Extrinsic factors include habitat loss, degradation and fragmentation, overexploitation, global climate change, diseases, environmental pollution, and biological invasion. Intrinsic factors include genetic diversity loss and inbreeding depression. Here, we only focus on three aspects that have shown significant progress in China over recent decades.

### Species endangerment and adaptation

Over the past decade, conservation genetics and genomics have evolved and progressed significantly in China. The genetic diversity, endangerment history and causes, and survival and adaptation strategies of many threatened species in China have been investigated, especially those of threatened vertebrates. This work has enabled the development of science-based conservation strategies. Two new sub-disciplines within conservation biology have been proposed recently. The first is conservation evolutionary biology [140]. The second is conservation metagenomics [141], based on the incorporation of evolutionary ideas and metagenomic technology.

#### Genetic diversity and evolutionary potential

High genetic diversity often implies high genetic evolutionary potential to respond to environmental changes. Genome-wide studies provide comprehensive insights into the evolutionary potential of threatened species. Recent studies revealed at least five cryptic phylogenetic species in Chinese giant salamanders (*Andrias davidianus*) [142] and two phylogenetic species in red pandas [143], which require subsequent lineage-specific conservation strategies. Consistent with their small population sizes, the threatened Baiji river dolphin, Myanmar snub-nosed monkey (*Rhinopithecus strykeri*), Himalayan red panda (*Ailurus fulgens*), ironwood tree (*Ostrya rehderiana*) and *Cercidiphyllum japonicum* [144] had very low genetic diversities [143,145,146]. Crested ibis (*Nipponia nippon*) populations have lost almost half of their ancestral genetic diversity [147]. These examples highlight the urgency of protecting their genetic diversity while simultaneously reducing the impacts of habitat degradation and human activities. In contrast, the threatened giant panda, Tibetan antelope (*Pantholops hodgsonii*) and Chinese red panda (*Ailurus styani*) still harbour high levels of genetic variation [71,143,148,149]. Thus, the assessment of genetic diversity can guide targeted conservation decisions.

#### Endangerment history and causes

Understanding the processes and drivers behind species endangerment are fundamental elements of effective wildlife conservation that genomic information can advance. For example, based on the whole diploid genome and population genome data, giant panda's evolutionary history traces back over eight million years. It included two population expansions, two bottlenecks and two divergences [148]. Further analysis revealed that Pleistocene climate changes and recent human activities were the major drivers of population fluctuations and divergences [148]. Interestingly, the golden snub-nosed monkey (*Rhinopithecus roxellana*) and Chinese red panda are largely sympatric in the Hengduan Mountains. They demonstrate a similar pattern of historical population fluctuation as the giant panda [143,150]. This pattern implies that Pleistocene climate changes might have affected the demographic histories of some sympatric mammals, especially large-bodied mammals, in similar ways. They may also reflect similar historical human pressures on these species.

In contrast, the narrowly distributed grey, black-and-white and Myanmar snub-nosed monkeys (*Rhinopithecus brelichi, R. bieti, R. strykeri*) and Himalayan red panda have experienced continuous population declines during the Pleistocene [143,150]. They display demographic histories different from the relatively widely distributed species above, highlighting the urgency to reduce genetic diversity loss in these species. For threatened plants, the widely distributed ginkgo's population genomics reconstructed multiple cycles of expansion and reduction during its evolutionary history [71]. In contrast, the critically endangered, narrowly distributed ironwood tree demonstrated continuous population decline during the Pleistocene [146]. Although the demographic patterns of widely and narrowly distributed threatened plants were similar to those of threatened mammals, the specific times of population expansion and reduction were different. Understanding the impact of climate change on different species and guilds, and using traits in addition to molecular data, may enable more effective management strategies to mitigate the future impacts of climate change on species diversity.

#### Survival and adaptation strategies

Species that survived through historical environmental change often evolved adaptive strategies to cope with the associated challenges. Recently, genome-wide studies have provided insights into the mechanisms of adaptive strategies in threatened species. For example, comparative genomics between the giant panda and the two red panda species revealed the genetic mechanisms underlying the morphological (pseudo-thumbs) and physiological convergence (specialized bamboo diet) [151]. The *DUOX2* gene's pseudogenization might be the genetic cause of the energy-saving low metabolic rate of giant pandas [152]. Genomic analysis found that in the high-altitude snub-nosed monkeys, some genes related to lung function, DNA repair and angiogenesis shared identical amino acid substitutions [153].

Besides the host genome itself, gut microbiota may also play an important role in the survival and adaptation of threatened species. Metagenomic analysis of gut microbiota identified crucial bacteria and digestive enzymes for helping digest cellulose and hemicellulose in the bamboo diet of giant pandas [154] and the leaf diet of golden snub-nosed monkeys [150]. They also help other species to adapt to the extreme environment of the Qinghai-Tibet Plateau [155].

### Biosafety and associated mechanisms

Biological invasions and transgenic crops are two important issues in biosafety. Both could greatly impact China's society, economy and biodiversity conservation and have been major topics of discussion within intergovernmental fora and agreements. In recent decades, as a response to technological advances and increasing capacity, the mechanisms, consequences and regulation of biological invasions and the benefits and adverse effects of transgenic crops have been well studied in China.

#### Biological invasions

The invasion success of alien species largely depends on species characteristics, ecosystem properties, biotic interactions and human activities [156–158]. In addition to life-history traits that link with invasive success, evolutionary or genomic characteristics are also important drivers. The genomic analysis of the widespread invasive plant species *Mikania micrantha* showed that half of its genome consists of long terminal-repeat retrotransposons, 80% of which derive from a significant expansion over the past 1 million years [157]. The evolutionary study of another highly invasive plant, *Ageratina adenophora*, suggests that it may have evolved to allocate more nitrogen to photosynthesis and reduce allocation to cell walls, resulting in stronger growth ability [159]. Notably, the nematode ascarosides’ pheromones play an important role in spreading pine-wilt disease [160]. The symbiotic microbes not only promote insect invasions but also coevolved with the invasive insects [158]. In addition, a global study of alien reptile and amphibian species suggests that both human-assisted dispersal and topographic heterogeneity increase the rate of spread for these species [156]. Species composition, phylogenetic and functional distances could also affect the invasion success [161].

Adverse consequences of these invasive species on biodiversity are widespread. The exotic *Spartina alterniflora* has homogenized the nematode communities in Chinese coastal wetland across different latitudes [162]. A global study of alien herpetofauna showed that biodiversity hotspots harbour more biological invasions than other regions [163]. Notably, a quantification for invasion risks of alien terrestrial vertebrates along China's Belt and Road Initiative countries suggests that 14 hotspots may be at particular risk of biological invasion [164]. These risks highlight the need for additional biosafety measures in these regions.

#### Impacts of transgenic crops

Transgenic crops could benefit people and nature by increasing food production per unit-area and decreasing chemical pesticide use. They also carry risks of harmful ecological impacts and present a significant threat to biodiversity [165,166]. A long-term (1990–2010) assessment of Bt cotton's widespread adoption and reduced insecticide use in northern China showed increases in abundance of three arthropod predators and decreases in aphid pests [167]. In contrast, this regional increase in Bt cotton adoption also progressively increased the population size of mirid bugs. They became pests in cotton and other crops [168]. One study considered the fitness effects of transgenic overexpression of a native gene (EPSP) developed to confer glyphosate resistance in rice. It found that transgenic F2 crop–weed hybrids produced 48%–125% more seeds per plant than non-transgenic controls in monoculture- and mixed-planting designs without glyphosate application. The findings suggest that over-expression of a native rice EPSP gene can lead to fitness advantages, even without glyphosate exposure. The over-expressed EPSP gene might result in fitness benefits in weedy relatives following transgene introgression [169]. However, little work has focused on the direct or indirect biodiversity effects of transgenic crops in China. Further work should develop better safeguards and better understand the impacts and trade-offs.

### Biodiversity responses to global change

Populations and species respond in various ways to global change, including shifts in abundances and geographic ranges, changes in phenology, behaviour and physiological plasticity, as well as evolutionary adaptations [170–173].

#### Species range shifts

In the face of a warming climate, species may move to higher latitudes or elevations to find a suitable habitat [171]. The large spans of latitude and elevation in China provide excellent opportunities to understand range shift dynamics. For example, the alpine treelines on the Qinghai-Tibetan Plateau have moved upward due to the climatic warming over the past century [174]. Species interactions, however, e.g. shrubby densification, may slow such movements [174]. Similarly, in recent decades hundreds of Chinese bird species with small body sizes, large geographical ranges, high trophic levels and high habitat specificity have moved to regions with higher elevation and latitude [175,176]. Studies have also reported range shifts in snakes, lizards and bats in the past few decades due to climate change in China [177–179]. A study using distribution data for 9701 plants across China examined the relationship between human activities and the degree to which species fill their potential climatic ranges [87]. Narrow-ranged and widespread species exhibited opposing responses to human activities. Human activities reduced ranges of narrow-ranged species, potentially threatening them. In contrast, human activities expanded ranges of widespread species, causing biotic homogenization [87].

#### Vegetation phenology change

Phenological change is another mechanism for adapting to climatic change. It includes the advance of spring phenology and delayed autumn phenology. These changes have been observed for trees, shrubs, herbs, insects and amphibians in China in the past half-century [180]. The high sensitivity of vegetation phenology to global change in the Qinghai-Tibetan Plateau has been studied in detail [181]. Specifically, growing seasons of trees are extending, showing earlier starts and later ends in response to climate warming based on tree-ring data from the Plateau [182].

In contrast, a study of meadow and steppe vegetation on the Plateau suggests that, although warm springs cause an advance in the start of the growing season, warm winters can also delay spring phases due to later fulfilment of chilling requirements. This results in delayed spring phenology [183]. Similarly, using observations on seven European tree species, it was demonstrated that the response of leaf unfolding to climate warming has significantly decreased in the past three decades, possibly linked to reduced chilling [184]. Notably, climate change effects on global vegetation phenology along elevational gradients are not consistent across different regions. This is potentially due to the differences in human disturbance, vegetation sensitivities to climate change and temperature lapse rates [185].

#### Behavioural and physiological plasticity and evolutionary adaptation

In addition to range shifts and phenological changes, species may also survive climate change through rapid behavioural or physiological plasticity or rapid evolutionary adaptation *in situ* [172]. An experimental study showed that even early-stage turtle embryos could move within the egg to adjust to small-scale thermal heterogeneity in their environment [186]. This thermoregulatory behaviour is widespread in reptile and bird species [187]. The embryo could also adjust its physiology to reduce the fitness penalties of adverse thermal conditions [172], as even small changes in egg temperature can impact offspring viability. Further, *Ciona savignyi*, an invasive marine tunicate, showed rapid adaptations to environmental changes through DNA methylation modification, causing significant epigenetic signatures [188].

## A PERSPECTIVE ON FUTURE BIODIVERSITY RESEARCH IN CHINA

Whilst there has been significant recent progress, considerable knowledge gaps remain. They include the geographic distributions and the conservation status of most species in China (e.g. small invertebrates, fishes, insects, bats, amphibians, reptiles), the role of trophic interactions (e.g. food webs and pollination networks) in biodiversity maintenance, the links between biodiversity and ecosystem services, and community-level responses to global change. The majority of the studies we have summarized focused on specific research questions in China. Many of these studies focused on application rather than conceptual or theoretical advances or methodological breakthroughs in basic biodiversity science. In the future, biodiversity research in China should also focus on fundamental scientific aspects to promote a deeper understanding of the problems. To achieve breakthroughs, we must identify priority topics and develop enabling conditions to meet these challenges. Such an approach should promote more China-led international collaborations to extrapolate results to the continental and global scale, and broader generalization and theory development. Below, we offer some general suggestions after considering practical guidance for scientists and policy-makers.

### Advancing priorities in Chinese biodiversity research

We selected four specific research fields which play critical roles in advancing our fundamental understanding about the origin, evolution and maintenance of biodiversity. First, as the Third Pole of the Earth, the Qinghai-Tibetan Plateau and the surrounding mountains serve as a perfect natural laboratory for biodiversity studies. There is some excellent research in this region (see our detailed review in section ‘Origin and evolution of China's biodiversity’). Nonetheless, there are still many research gaps and areas with relatively little survey data. Examples include the southern slope of the Himalayas and the high mountains and valleys of the region. Moreover, recent global climate and land-use changes are dramatically changing the environment of the Qinghai-Tibetan Plateau [128]. Therefore, further monitoring of the environment is needed to protect its biodiversity and fragile ecosystems more effectively.

Second, subtropical forests in China cover over 25% of the total land area (Fig. 2), ranging from 23ºN to 33ºN in latitude and 93ºE to 123ºE in longitude. China harbours nearly 70% of the total global area of subtropical forest, while other regions at these latitudes are deserts or semi-deserts [189,190]. Most subtropical forests are evergreen broadleaved, with high levels of biodiversity and endemism. Over one-third of China's vascular plant species and nearly 35% of national nature reserves are in this region [189]. Various biodiversity and ecological studies have been carried out in these regions [13,47,69,119]. Further studies should address both the serious anthropogenic threats by intensive agriculture and industrial activities and the high degrees of urbanization, and the importance of subtropical forests for biodiversity globally and ecosystem functioning and services at local to regional levels [189].

Third, with 32 000 km of coastline, China's seas cover an area of 4.73 million km^2^ [9]. The last 70 years have seen significant advances in marine biodiversity research [9]. Nonetheless, there is still a large disparity in marine biodiversity studies’ advancement compared to terrestrial biodiversity studies. Further studies in marine biodiversity should strengthen marine specimens’ collections, monitor marine biodiversity in various habitats, and conduct research on biodiversity changes in Chinese seas and nearshore ecosystems. There should be standard monitoring practices to assess change over time [9]. The recent advances in terrestrial biodiversity studies also provide excellent examples to develop marine biodiversity monitoring networks. Coastal ecosystems are also under-researched. They include some of the most threatened ecosystems in terms of loss, degradation and reclamation [191].

Fourth, as the world's most populous country with a long history of human activity, China faces a severe challenge to balance biodiversity and socio-economic development, especially in eastern regions with intensive human activities [87]. China also provides a key area for understanding how to conserve and restore biodiversity in the Anthropocene. That understanding must include the effects of rapid urbanization on ecological community assembly and plant–animal interactions [192], effects of climate change on the phenology and demography of species [181], and the basis for rewilding or reintroducing extirpated species back into natural areas [193]. The Yangtze River is the third-longest river in the world. The surrounding regions still face severe threats to freshwater and terrestrial biodiversity due to rapid urbanization in the delta and the construction of dams along the river. Dams causing waterway fragmentation within China have reduced biodiversity both above and below the dams and reduced genetic connectivity by impairing dispersal and migration of freshwater organisms [194]. Although the Chinese national government and scientists are making considerable efforts to monitor and protect this region's biodiversity, there is still a long way to go. Calls for more systematic planning of river-basin management have been slow to be enacted. Effective monitoring of biodiversity changes is urgently needed, in addition to exploring the underlying mechanisms of species coexistence, quantifying threats to endangered species and evaluating the synergism between biodiversity and socio-economic development.

### Translating scientific advances into biodiversity conservation and ecosystem restoration practices

Ecological civilization is an explicit national governance strategy of the Chinese government to prioritize the protection and restoration of China's biodiversity. It includes the development and implementation of the ecological redline policy [195], the construction of a national-park-centred protected-area system, and the recent enforcement of the permanent ban on wildlife trade and consumption. The ecological conservation redline policy aims to include a quarter of China's land to protect most species and their habitats in China [195]. China's updated national park system was proposed in 2013. It builds the system's basic framework through 10 pilot national parks of over 220 000 km^2^ [196]. Recent advances in identifying biodiversity hotspots, species distributions, drivers of threat and habitat fragmentation provide a robust scientific basis for selecting the areas for ecological redlines and designing and managing national parks [13,197]. Research on mechanisms behind biodiversity and the relationship between biodiversity and ecosystem functioning facilitates the conservation and restoration of endangered species and degraded ecosystems [110,121]. At present, the connections between scientists, policy-makers, stakeholders and the public are frequently weak. Communication between scientists with the public should be improved. For example, using citizen scientists to collect biodiversity data is pivotal in addressing gaps in biodiversity targets. To establish better collaboration with policy-makers to enable science-based policy and implementation, the China Council for International Cooperation on Environment and Development (CCICED) has initiated special policy studies which work alongside China's five-year plans (http://www.cciced.net/cciceden/).

### Advanced technology application in biodiversity research

With further innovations in genomics, remote sensing and other technologies, we can expect to see additional complementary and synergistic research that uses China's continually expanding research capacity to further push the research frontiers in biodiversity science.

The rapid development of high-throughput sequencing technology and genomics has promoted insights into species’ genetic diversity, from several molecular markers to the whole-genome level. The decrease of sequencing costs enables large-scale genomic diversity surveys at the species level. Chinese scientists and institutions have played an indispensable role in this genomics wave. For example, in 2010, international scientists launched a G10K genome project to sequence the genomes of 10 000 vertebrate species. The Beijing Genomics Institute-Shenzhen played an important role, including leading the One Thousand Plants and Animals Genome Project (2010), the One Thousand Insects Transcriptome Project (1KITE, 2011) and the Ten Thousand Birds Genome Project (B10K, 2014). Recently, research teams from the Chinese Academy of Sciences also led the Ten Thousand Fishes Genome Project (2019) and the Ten Thousand Protists Genome Project (2019). These massive genome projects have provided unprecedented insights into genomic diversity and enabled a more in-depth understanding of biodiversity evolution. In addition, the rapid application of high-throughput sequencing technology has also facilitated the development of the International Barcode of Life (iBOL) project. Chinese scientists have substantially contributed to the iBOL project, especially for insects and vascular plants [198]. With further advancement of these approaches, we can expect to transform our understanding of various evolutionary processes of biodiversity on regional and global scales.

Advances in monitoring techniques have allowed an understanding of the factors driving generalization and specialization from the cellular and molecular level to the factors governing community composition, ecosystem functioning and ecosystem service provision. Advanced remote sensing technologies allow almost real-time spatially continuous large-scale monitoring of ecosystems using different sensors to explore leaf chemical composition, biodiversity and functional traits [199,200]. China has the technology to implement these approaches, but frameworks to promote more interdisciplinary research are still needed. Long-term biodiversity monitoring using traditional and novel methods is the key to addressing fundamental and practical ecology questions. Scientists across China have established several long-term biodiversity monitoring networks. By taking advantage of the extensive coverage and uniqueness of China's ecosystems and biodiversity, we believe that coordinated, distributed experiment and observation networks with long-term monitoring will substantially improve research infrastructure and quality. Interdisciplinary facilitation mechanisms to enhance cooperation between groups and institutions are also required to enable cross-taxa research. The development of standards and protocols must ensure the collection and dissemination of biodiversity data in a coherent and standardized way to enable better comparisons between and within sites over time. China's extensive biodiversity science research is starting to provide long-term data on numerous ecosystems across space and time. However, data collection standards and approaches and data-sharing mechanisms will need further attention to improve analyses. Increasing interactions with global networks have enabled global standards for field census data, but such standards are less frequent for other biodiversity data. Thus, further collaborative actions, shared sites and coordination must ensure that the huge effort going into biodiversity research within China can be entirely complementary.

### Strengthening and expanding international collaborations

Solving many questions in biodiversity requires global collaboration. Chinese scientists have greatly benefited from close cooperation with foreign scientists and international organizations in the last few decades. However, further partnerships are needed to maintain and strengthen the existing joint research projects, support the ongoing establishment of regional collaborations (e.g. Mapping Asia Plants Project, Sino-Africa Joint Research Center, and Southeast Asia Biodiversity Research Institute), and develop new cooperative biodiversity research platforms. The recent development of the Belt and Road Initiative (BRI), initialized by the Chinese government in 2013, provides the opportunity to expand the collaborative research in biodiversity between China and over 60 countries across mainland Eurasia, Africa and the Middle East [201] alongside the development of linear infrastructure across these countries. The Belt and Road science plan aims to overcome some of the challenges associated with developing sustainably at an unprecedented scale through the use of big data and remote sensing, coupled with capacity-building initiatives. Attention is needed to ensure that the route itself avoids the key biodiversity areas. Moreover, supportive infrastructure and raw materials also need to be accounted for to prevent a much wider footprint than the new linear infrastructure alone will cause. New cooperative mechanisms need to reduce the potential risks of the BRI on biodiversity, such as habitat fragmentation and biological invasions [201].

Meanwhile, China harbours several international biodiversity hotspots which cross national boundaries, such as the Himalaya, Mountains of Central Asia, and Indo-Burma. Transboundary cooperation through coordinated inventory, monitoring and research needs to be strengthened substantially to protect these key areas [202]. They include populations of endangered species, such as the critically endangered Amur leopards across China and Russia [203]. Chinese biodiversity researchers also need to become more active in global intergovernmental initiatives with relevance to biodiversity, such as IPBES (the Intergovernmental Science-Policy Platform on Biodiversity and Ecosystem Services), CBD (Convention on Biological Diversity), CITES (the Convention on International Trade in Endangered Species of Wild Fauna and Flora) and IPCC (Intergovernmental Panel on Climate Change), and make greater contributions to the UN Sustainable Development Goals (SDGs).

In sum, given the rapid evolution of biodiversity research within China and increasing international leadership, China's biodiversity research has a bright future. New syntheses between these different research topics and deeper collaborations across different disciplines will advance our understanding of biodiversity's patterns and processes. Eventually, it will help build ‘a shared future for all life on Earth’.

## Supplementary Material

nwab032_supplemental_file
