# Supplementary material for: The global significance of biodiversity science in China: an overview
Source: Natl Sci Rev. 2021 Feb 23;8(7):nwab032. doi: 10.1093/nsr/nwab032 (PMC8310773; doi:10.1093/nsr/nwab032)
Supplement: nwab032_supplemental_file [file nwab032_supplemental_file.docx]

**Supplementary materials**

**The global significance of biodiversity science in China: an overview**

Xiangcheng Mi, Gang Feng, Yibo Hu, Jian Zhang, Lei Chen, Richard T. Corlett, Alice C. Hughes, Stuart Pimm, Bernhard Schmid, Suhua Shi, Jens-Christian Svenning, Keping Ma

**Table S1 List of Volumes for Flora Republicae Popularis Sinicae**

1. Wu, Z. Volume 1: Introduction, Tetracentraceae. Beijing: Science Press; 2004.
2. Qin, R. Volume 2: Ophiogiossaceae-Oleandra. Beijing: Science Press; 1959.
3. Qin, R. Volume 3 (1): Acrostichaceae, Adiantaceae, Hemionitidaceae, Parkeriacae, Pteridaceae, Pteridiaceae, Sinopteridaceae, Stenochlanaceae. Beijing: Science Press; 1990.
4. Zhu, W. Volume 3 (2): Antrophyaceae, Vittariaceae, Atryriaceae. Beijing: Science Press; 1999.
5. Xing, G. Volume 4 (1): Hypodematiaceae, Thelypteridaceae. Beijing: Science Press; 1999.
6. Wu, Z. Volume 4 (2): Aspleniaceae, Pleurosoriopsidaceae, Onocleaceae, Woodsiaceae, Blechnacea, Peranemaceae. Beijing: Science Press; 1999.
7. Wu, S. Volume 5 (1): Dryopteridaceae (1). Beijing: Science Press; 2000.
8. Kong, X. Volume 5 (2): Dryopteridaceae (2). Beijing: Science Press; 2001.
9. Wu, Z. Volume 6 (1): Aspidaceae, Bolbitidaceae, Lomariopsidaceae, Elaphoglossaceae, Nephrolepidaceae, Oleandraceae, Davalliaceae, Gymnogrammitidaceae. Beijing: Science Press; 1999.
10. Lin, Y. Volume 6 (2): Dipteridaceae, Cheiropleuriacae, Polypodiaceae, Drynariaceae, Platyceriaceae, Grammtidaceae, Loxogrammaceae, Marsileaceae, Salviniaceae, Azollaceae. Beijing: Science Press; 2000.
11. Zhang, X. Volume 6 (3): Huperziaceae, Lycopodiaceae, Selaginellaceae, Isoetaceae, Equisetaceae, Psilotaceae, Marattiaceae, Cyatheaceae. Beijing: Science Press; 2004.
12. Zheng, W and Fu, L. Volume 7: Araucaruaceae, Cephalotaxaceae, Cycadaceae, Cupressaceae, Ephedraceae, Ginkgoaceae, Gnetaceae, Pinaceae, Podocarpaceae, Taxaceae, Taxodiaceae. Beijing: Science Press; 1978.
13. Sun, X. Volume 8: Alismataceae, Apongetonaceae, Butomaceae, Hydrocharitaceae, Najadaceae, Pandanaceae, Potamogetonaceae, Scheuchzeriaceae, Sparganiaceae, Triuridaceae, Typhaceae. Beijing: Science Press; 1992.
14. Geng, B, Wang, Z. Volume 9 (1): Gramineae (1): Bambusoideae. Beijing: Science Press; 1996.
15. Liu, L. Volume 9 (2): Gramineae (2). Beijing: Science Press; 2002.
16. Guo, B. Volume 9 (3): Gramineae (3). Beijing: Science Press; 1987.
17. Chen, S. Volume 10 (1): Gramineae (4). Beijing: Science Press; 1990.
18. Chen, S. Volume 10 (2): Gramineae (5). Beijing: Science Press; 1997.
19. Tang, J and Wang, F. Volume 11: Cyperaceae (1): Scirpus-Scleria. Beijing: Science Press; 1961.
20. Dai, L and Liang, S. Volume 12: Cyperaceae (2): Caricoideae. Beijing: Science Press; 2000.
21. Pei, S and Chen, S. Volume 13 (1): Palmae. Beijing: Science Press; 1991.
22. Wu, G. Volume 13 (3): Flagellariaceae, Commenlinaceae, Restionaceae, Pontederiaceae, Centrolepidaceae. Beijing: Science Press; 1997.
23. Wu, Z and Li, H. Volume 13 (2): Araceae, Lemnaceae. Beijing: Science Press; 1978.
24. Tang, J and Wang, F. Volume 14: Liliaceae (1). Beijing: Science Press; 1980.
25. Tang, J and Wang, F. Volume 15: Liliaceae (2). Beijing: Science Press; 1978.
26. Pei, J and Ding, Z. Volume 16 (1): Amaryllidaceae, Dioscoreaceae, Iridaceae, Taccaceae. Beijing: Science Press; 1985.
27. Wu, D. Volume 16 (2): Burmanniaceae, Cannaceae, Marantaceae, Musaceae, Zingiberaceae. Beijing: Science Press; 1981.
28. Lang, K. Volume 17: Orchidaceae (1). Beijing: Science Press; 1999.
29. Chen, X. Volume 18: Orchidaceae (2). Beijing: Science Press; 1999.
30. Ji, Z. Volume 19: Orchidaceae (3). Beijing: Science Press; 1999.
31. Cheng, Y. Volume 20 (1): Casuarinaceae Chloranthaceae, Piperaceae, Saururaceae. Beijing: Science Press; 1982.
32. Wang, Z and Fang, Z. Volume 20 (2): Salicaceae. Beijing: Science Press; 1984.
33. Kuang, K and Li, P. Volume 21: Betulaceae, Juglandaceae, Myricaceae. Beijing: Science Press; 1979.
34. Chen, H and Huang, C. Volume 22: Fagaceae, Ulmaceae, Rhoipteleaeae. Beijing: Science Press; 1998.
35. Zhang, X and Wu, Z. Volume 23 (1): Moraceae. Beijing: Science Press; 1998.
36. Wang, W and Chen, J. Volume 23 (2): Urticaceae. Beijing: Science Press; 1995.
37. Qiu, H and Lin, Y. Volume 24: Aristolochiaceae, Balanophoraceae, Loranthaceae, Olacaceae, Opiliaceae, Podostemaceae, Proteaceae, Rafflesiaceae, Santalaceae. Beijing: Science Press; 1988.
38. Li, A. Volume 25 (1): Polygonaceae 1998.
39. Kong, X. Volume 25 (2): Amaranthceae, Chenopodiaceae. Beijing: Science Press; 1979.
40. Tang, C. Volume 26: Angiospermae, Dicotyledoneae, Nyctaginaceae, Portulacaceae, Phytolaccaceae, Basellaceae, Aizoaceae, Caryophyllaceae. Beijing: Science Press; 1996.
41. Guan, K. Volume 27: Ceratophyllaceae, Cercidiphyllaceae, Eupteleaceae, Nymphaeceae, Ranunculaceae (1), Trochodendraceae. Beijing: Science Press; 1979.
42. Wang, W. Volume 28: Ranunculaceae (2). Beijing: Science Press; 1980.
43. Ying, J and Chen, D. Volume 29: Lardizabalaceae, Berberidaceae. Beijing: Science Press; 2001.
44. Liu, Y. Volume 30 (1): Menispermaceae, Magnoliaceae. Beijing: Science Press; 1996.
45. Jiang, Y and Li, B. Volume 30 (2): Annonaceae, Calycanthaceae, Myristicaceae. Beijing: Science Press; 1979.
46. Li, X. Volume 31: Hernandiaceae, Lauraceae. Beijing: Science Press; 1982.
47. Wu, Z. Volume 32: Papaveraceae, Capparaceae. Beijing: Science Press; 1999.
48. Zhou, T. Volume 33: Crucifereae. Beijing: Science Press; 1987.
49. Fu, S and Fu, K. Volume 34 (1): Bretschneideraceae, Crassulaceae, Droseraceae, Moringaceae, Nepenthaceae, Resedaceae. Beijing: Science Press; 1984.
50. Pan, J. Volume 34 (2): Saxifragaceae (1). Beijing: Science Press; 1992.
51. Lu, L and Huang, S. Volume 35 (1): Saxifragaceae (2). Beijing: Science Press; 1995.
52. Zhang, H. Volume 35 (2): Eucommiaceae, Hamamelidaceae, Pittosporaceae, Platanaceae. Beijing: Science Press; 1979.
53. Yu, D. Volume 36: Rosaceae (1). Beijing: Science Press; 1974.
54. Yu, D. Volume 37: Rosaceae (2). Beijing: Science Press; 1985.
55. Yu, D. Volume 38: Connaraceae, Rosaceae (3). Beijing: Science Press; 1986.
56. Chen, D. Volume 39: Leguminosae (1). Beijing: Science Press; 1988.
57. Wei, Z. Volume 40: Leguminosae (2): Faboideae (1). Beijing: Science Press; 1994.
58. Li, S. Volume 41: Leguminosae (3): Faboideae (2). Beijing: Science Press; 1995.
59. Fu, K. Volume 42 (1): Leguminosae (4). Beijing: Science Press; 1993.
60. Cui, H. Volume 42 (2): Leguminosae (5). Beijing: Science Press; 1998.
61. Xu, L and Huang, C. Volume 43 (1): Oxalidaceae, Geraniaceae, Tropaeolaceae, Linaceae, Erythroxylaceae, Zygophyllaceae. Beijing: Science Press; 1998.
62. Huang, C. Volume 43 (2): Rutaceae. Beijing: Science Press; 1997.
63. Chen, S. Volume 43 (3): Burseraceae, Dichapetalaceae, Malpighiaceae, Polygalaceae, Simaroubaceae. Beijing: Science Press; 1997.
64. Li, B. Volume 44 (1): Euphorbiaceae (1): Phyllanthoideae. Beijing: Science Press; 1994.
65. Qiu, H. Volume 44 (2): Euphorbiaceae (2): Acalyphoideae, Crotonoideae. Beijing: Science Press; 1996.
66. Ma, J. Volume 44 (3): Euphorbioideae (3). Beijing: Science Press; 1997.
67. Zheng, M and Min, T. Volume 45 (1): Anacardiaceae, Buxaceae, Callitrichaceae, Coriaceae, Daphniphyllaceae, Empetraceae, Pentaphylacaceae. Beijing: Science Press; 1980.
68. Chen, S. Volume 45 (2): Aquifoliaceae. Beijing: Science Press; 1999.
69. Cheng, J and Huang, P. Volume 45 (3): Celastraceae. Beijing: Science Press; 1999.
70. Fang, W. Volume 46: Aceraceae, Hippocastanaceae, Hippocrateaceae, Icacinaceae, Salvadoraceae, Staphyleaceae. Beijing: Science Press; 1981.
71. Liu, H and Luo, X. Volume 47 (1): Sabiaceae, Sapindaceae. Beijing: Science Press; 1985.
72. Chen, Y. Volume 47 (2): Balsaminaceae. Beijing: Science Press; 2002.
73. Chen, Y. Volume 48 (1): Rhamnaceae. Beijing: Science Press; 1982.
74. Li, C. Volume 48 (2): Vitaceae. Beijing: Science Press; 1998.
75. Zhang, H and Miao, R. Volume 49 (1): Elacocarpaceae, Tiliaceae. Beijing: Science Press; 1989.
76. Feng, G. Volume 49 (2): Actinidiaceae, Bombacaceae, Dilleniaceae, Malvaceae, Ochnaceae, Sterculiaceae. Beijing: Science Press; 1984.
77. Zhang, H. Volume 49 (3): Theaceae (1): Theoideae. Beijing: Science Press; 1998.
78. Lin, L. Volume 50 (1): Theaceae (2): Ternstroemoideae. Beijing: Science Press; 1998.
79. Li, X. Volume 50 (2): Bixaceae, Cistaceae, Dipterocarpaceae, Elatinaceae, Frankeniaceae, Guttiferae, Tamaricaceae. Beijing: Science Press; 1990.
80. Wang, Q. Volume 51: Violaceae. Beijing: Science Press; 1991.
81. Gu, C. Volume 52 (1): Flacourtiaceae, Stachyuraceae, Passifloraceae, Caricaceae, Begoniaceae, Tetramelaceae, Thymelaeaceae, Cactaceae, Ancistrocladaceae. Beijing: Science Press; 1999.
82. Fang, W. Volume 52 (2): Alangiaceae, Crypteroniaceae, Elaeagnaceae, Lecythidaceae, Lythraceae, Nyssaceae, Punicaceae, Rhizophoraceae, Sonneratiaceae. Beijing: Science Press; 1983.
83. Chen, J. Volume 53 (1): Combretaceae, Melastomataceae, Myrtaceae. Beijing: Science Press; 1984.
84. Chen, J. Volume 53 (2): Trapaceae, Onagraceae, Haloragidaceae, Hippuridaceae, Theligonaceae, Cynomoriaceae. Beijing: Science Press; 2000.
85. He, J and Zeng, C. Volume 54: Araliaceae. Beijing: Science Press; 1978.
86. Shan, R and She, M. Volume 55 (1): Umbelliferae (1). Beijing: Science Press; 1979.
87. Shan, R and She, M. Volume 55 (2): Umbelliferae (2). Beijing: Science Press; 1985.
88. Shan, R and She, M. Volume 55 (3): Umbelliferae (3). Beijing: Science Press; 1992.
89. Fang, W and Hu, W. Volume 56: Clethraceae, Cornaceae, Diapensiaceae, Pyrolaceae. Beijing: Science Press; 1990.
90. Fang, R. Volume 57 (1): Ericaceae (1): Rhododendroideae. Beijing: Science Press; 1999.
91. Hu, L, Fang, M. Volume 57 (2): Ericaceae (2). Beijing: Science Press; 1994.
92. Fang, R. Volume 57 (3): Ericaceae (3). Beijing: Science Press; 1991.
93. Chen, J. Volume 58: Myrsinaceae. Beijing: Science Press; 1979.
94. Chen, F and Hu, Q. Volume 59 (1): Primulaceae (1): Lpsimachieae – Primuleae. Beijing: Science Press; 1989.
95. Chen, F and Hu, Q. Volume 59 (2): Primulaceae (2): Primuleae - Samoleae. Beijing: Science Press; 1990.
96. Li, S. Volume 60 (1): Ebenaceae, Plumbaginaceae, Sapotaceae. Beijing: Science Press; 1987.
97. Wu, R and Huang, S. Volume 60 (2): Styracaceae, Symplocaceae. Beijing: Science Press; 1987.
98. Zhang, M and Qiu, L. Volume 61: Loganiaceae, Oleaceae. Beijing: Science Press; 1992.
99. He, T. Volume 62: Gentianaceae. Beijing: Science Press; 1988.
100. Jiang, Y and Li, B. Volume 63: Apocynaceae, Asclepiadaceae. Beijing: Science Press; 1977.
101. Wu, Z. Volume 64 (1): Convolvulceae, Hydrophyllaceae, Polemoniaceae. Beijing: Science Press; 1979.
102. Kong, X, Wang, W. Volume 64 (2): Boraginaceae. Beijing: Science Press; 1989.
103. Pei, J and Chen, S. Volume 65 (1): Verbenaceae. Beijing: Science Press; 1982.
104. Wu, Z and Li, X. Volume 65 (2): Labiatae (1). Beijing: Science Press; 1977.
105. Wu, Z and Li, X. Volume 66: Labiatae. Beijing: Science Press; 1977.
106. Kuang, K and Lu, A. Volume 67 (1): Solanaceae. Beijing: Science Press; 1978.
107. Zhong, B. Volume 67 (2): Scrophulariaceae (1). Beijing: Science Press; 1963.
108. Zhong, B. Volume 68: Scrophulariaceae (2). Beijing: Science Press; 1963.
109. Wang, W. Volume 69: Bignoniaceae, Gesneriaceae, Lentibulariaceae, Martyniaceae, Orobanchaceae, Pedaliaceae. Beijing: Science Press; 1990.
110. Hu, J. Volume 70: Acanthaceae, Myoporaceae, Phrymaceae, Plantaginaceae. Beijing: Science Press; 2002.
111. Luo, X. Volume 71 (1): Rubiaceae (1). Beijing: Science Press; 1999.
112. Chen, W. Volume 71 (2): Rubiaceae (2): Rubioideae. Beijing: Science Press; 1999.
113. Xu, B. Volume 72: Caprifoliaceae. Beijing: Science Press; 1988.
114. Lu, A and Chen, S. Volume 73 (1): Adoxaceae, Cucurbitaceae, Dipsacaceae, Valeruianaceae. Beijing: Science Press; 1986.
115. Hong, D. Volume 73 (2): Campanulaceae, Goodeniaceae, Stylidiaceae. Beijing: Science Press; 1983.
116. Lin, R and Chen, Y. Volume 74: Compositae (1): Vernonieae - Astereae. Beijing: Science Press; 1985.
117. Lin, R. Volume 75: Compositae (2): Inuleae - Helenieae. Beijing: Science Press; 1979.
118. Lin, R and Shi, Z. Volume 76 (1): Compositae (3): Anthemideae (1). Beijing: Science Press; 1983.
119. Lin, R and Lin, Y. Volume 76 (2): Compositae (4): Anthemideae (2). Beijing: Science Press; 1991.
120. Chen, Y. Volume 77 (1): Compositae (5): Senecioneae, Caleneuleae. Beijing: Science Press; 1999.
121. Lin, R and Liu, S. Volume 77 (2): Compositae (6): Senecioneae, Senecioninae. Beijing: Science Press; 1987.
122. Lin, R and Shi, Z. Volume 78 (1): Compositae (7): Echinopsideae, Cynareae. Beijing: Science Press; 1987.
123. Chen, Y and Shi, Z. Volume 78 (2): Compositae (8): Cynareae. Beijing: Science Press; 1999.
124. Cheng, Y. Volume 79: Compositae (9): Mutisieae. Beijing: Science Press; 1996.
125. Lin, R and Shi, Z. Volume 80 (1): Compositae (10): Cichorioideae, Cichorieae. Beijing: Science Press; 1997.
126. Ge, X and Lin, Y. Volume 80 (2): Compositae (11): Cichorieae, Taraxacum. Beijing: Science Press; 1999.

**Table S2 List of Volumes for Fauna Sinicae**

1. **Mammals**
2. Luo, Z, Chen , W and Gao, W. Mammalia, Volume 6: Rodentia Part III: Cricetidae. Beijing: Science Press; Beijing: Science Press. Beijing: Science Press; 2000.
3. Gao, Y, Wang, S, Wang, S, et al. Mammalia, Volume 8: Carnivora. Beijing: Science Press; Beijing: Science Press. Beijing: Science Press; 1987.
4. Zhou, K. Mammalia, Volume 9: Cetacea: Carnivora: Phocoidea: Sirenia. Beijing: Science Press; 2004.
5. **Birds**
6. Zheng, Z, Zheng, G and Zhang, F, et al. Aves, Volume 1: Part 1 (Introductory Account of the Class Aves in China) and Part 2 (Account of Orders listed in this Volume) 1997.
7. Zheng, Z. Aves, Volume 2: Anseriformes. Beijing: Science Press; 1979.
8. Zheng, Z. Aves, Volume 4: Galliformes. Beijing: Science Press; 1978.
9. Wang, Q, Ma, M and Gao, Y. Aves, Volume 5: Gruiformes, Charadriiformes and Lariformes. Beijing: Science Press; 2006.
10. Zheng, Z, Xian, Y and Guan, G. Aves, Volume 6: Columbiformes, Psittaciformes, Cucliformes and Strigiformes. Beijing: Science Press; 1991.
11. Tan, Y and Guan, G. Aves, Volume 7: Caprimulgiformes, Apodiformes, Trogoniformes, Coraciiformes and Piciformes. Beijing: Science Press; 2003.
12. Zheng, Bea. Aves, Volume 8: Passeriformes: Eurylaimidae - Irenidae. Beijing: Science Press; 1985.
13. Chen, F and Luo, S. Aves, Volume 9: Passeriformes: Bombycillidae - Prunellidae. Beijing: Science Press; 1998.
14. Zheng, Z, Long, Z and Lu, T. Aves, Volume 10: Passeriformes: Muscicapidae 1, Turdidae. Beijing: Science Press; 1995.
15. Zheng, Z, Long, Z and Zheng, B. Aves, Volume 11: Passeriformes: Muscicapidae 2, Timaliinae. Beijing: Science Press; 1987.
16. Zheng, Z, Lu, T and Yang, L, et al. Aves, Volume 12: Passeriformes: Muscicapidae 3, Sylvinae, Muscicapinae 2010.
17. Li, G, Zheng, B and Liu, G. Aves, Volume 13: Passeriformes: Paridae - Zosteropidae. Beijing: Science Press; 1982.
18. Fu, T, Song, Y and Gao, W. Aves, Volume 14: Passeriformes: Ploceidae - Fringillidae. Beijing: Science Press; 1998.
19. **Reptiles**
20. Zhang, M, Zong, Y and Ma, J. Reptilia, Volume 1: General Accounts of Reptilia, Testudoformes and Crocodiliformes. Beijing: Science Press; 1998.
21. Zhao, E, Zhao, K and Zhou, Kea. Reptilia, Volume 2: Squamata: Lacertilia. Beijing: Science Press; 1999.
22. Zhao, E and Huang, M, Zong, Y. Reptilia, Volume 3: Squamata: Serpentes. Beijing: Science Press; 1998.
23. **Amphibians**
24. Fei, L and Hu, S. Amphibia, Volume 1: General Account of Amphibia: Gymnophiona and Urodela. Beijing: Science Press; 2006.
25. Fei, L, Hu, S and Ye, C, et al. Amphibia, Volume 2: Anura. Beijing: Science Press; 2009.
26. Fei, L, Hu, S and Ye, C, et al. Amphibia, Volume 3: Anura: Ranidae. Beijing: Science Press; 2009.
27. **Osteichthyes and Cyclostomata**
28. Li, S and Wang, H. Osteichthyes: Pleuronectiformes. Beijing: Science Press; 1995.
29. Su, J and Li, C. Osteichthyes: Tetraodontiformes, Pegasiformes, Gobiesociformes, Lophiiformes. Beijing: Science Press; 2002.
30. Chen, Y. Osteichthyes: Cypriniformes II. Beijing: Science Press; 1998.
31. Chu, X and Zheng, B, Dai, D. Osteichthyes: Siluriformes. Beijing: Science Press; 1999.
32. Yue, P. Osteichthyes: Cypriniformes III. Beijing: Science Press; 2000.
33. Zhang, S. Osteichthyes: Acipenseriformes, Elopiformes, Clupeiformes, Gonorhynchiformes. Beijing: Science Press; 2001.
34. Chen, S. Oteichthyes: Myctophiformes, Cetomimiformes, Osteolossiformes. Beijing: Science Press; 2002.
35. Jin, X. Osteichthyes: Scorpaeniformes. Beijing: Science Press; 2006.
36. Wu, H and Zhong, J. Osteichthyes: Perciformes (V) Gobioidei. Beijing: Science Press; 2008.
37. Zhang, C. Osteichthyes: Anguilliformes, Notacanthiformes. Beijing: Science Press; 2010.
38. Li, S and Zhang, C. Osteichthyes: Atheriniformes, Cyprinodontiformes, Beloniformes, Ophidiiformes, Gadiformes. Beijing: Science Press; 2011.
39. Liu, J. Osteichthyes: Perciformes IV. Beijing: Science Press; 2016*.
40. Zhu, Y and Meng, Q. Cyclostomata, Chondrorichthyes. Beijing: Science Press; 2001.

**6. Invertebrata**

1. Jiang, X and Du, N. Invertebrata, Volume 1: Crustacea: Freshwater Cladocera. Beijing: Science Press; 1979.
2. Shen, J. Invertebrata, Volume 2: Crustacea: Freshwater Copepoda. Beijing: Science Press; 1979.
3. Chen, X. Invertebrata, Volume 3: Platyhelminthes, Trematoda: Digenea (I). Beijing: Science Press; 1985.
4. Dong, Z. Invertebrata, Volume 4: Mollusca: Cephalopode. Beijing: Science Press; 1988.
5. Yang, T. Invertebrata, Volume 5: Annelida: Hirudinea. Beijing: Science Press; 1996.
6. Liang, Y. Invertebrata, Volume 6: Echinodermata: Holothuroidea. Beijing: Science Press; 1997.
7. Ma, X. Invertebrata, Volume 7: Mollusca: Gastropoda: Mesogastropoda: Cypraeacea. Beijing: Science Press; 1997.
8. Song, D and Zhu, M. Invertebrata, Volume 8: Arachnida: Araneae: Thomisidae: Philodromidae. Beijing: Science Press; 1997.
9. Wu, B, Wu, Q and Qiu, J, et al. Invertebrata, Volume 9: Annelida: Polychaeta (I): Phyllodocimorpha. Beijing: Science Press; 1997.
10. Yin, C. Invertebrata, Volume 10: Arachnida: Araneae: Araneidae. Beijing: Science Press; 1997.
11. Lin, G. Invertebrata, Volume 11: Mollusca: Gastropoda: Cephalaspidea. Beijing: Science Press; 1997.
12. Wang, Z. Invertebrata, Volume 12: Mollusca: Bivalvia: Mytiloida. Beijing: Science Press; 1997.
13. Zhu, M. Invertebrata, Volume 13: Arachnida: Araneae: Theridiidae. Beijing: Science Press; 1998.
14. Tan, Z. Invertebrata, Volume 14: Protozoa: Sacodina: Acantharia, Spumellaria. Beijing: Science Press; 1998.
15. Chen, Q and Ma, C. Invertebrata, Volume 15: Myxozoa: Myxosporea. Beijing: Science Press; 1998.
16. Pei, Z. Invertebrata, Volume 16: Coelenterata: Anthozoa: Actiniaria, Ceriantharia, Zoanthidea. Beijing: Science Press; 1998.
17. Dai, A. Invertebrata, Volume 17: Arthropoda: Crustacea: Malacostraca: Decapoda: Parathelphusidae, Potamidae. Beijing: Science Press; 1999.
18. Yin, W. Invertebrata, Volume 18: Arthropoda: Protura. Beijing: Science Press; 1999.
19. Chen, D and Zhang, G. Invertebrata, Volume 19: Mollusca: Gastropoda: Stylommatophora: Clausiliidae. Beijing: Science Press; 1999.
20. Xu, F. Invertebrata, Volume 20: Mollusca: Bivalvia: Protobranchia, Anomalodesmata. Beijing: Science Press; 1999.
21. Liu, R and Wang, S. Invertebrata, Volume 21: Arthropoda: Crustacea: Malacostraca: Mysidacea. Beijing: Science Press; 2000.
22. Wu, B, Lang, S and Wang, Wea. Invertebrata, Volume 22: Platyhelminthes: Monogenea. Beijing: Science Press; 2000.
23. Zou, R. Invertebrata, Volume 23: Coelenterata: Anthozoa: Scleractinia: Hermatypic Coral. Beijing: Science Press; 2001.
24. Zhuang, Q. Invertebrata, Volume 24: Mollusca: Bivalvia: Veneridae. Beijing: Science Press; 2001.
25. Wu, S. Invertebrata, Volume 25: Nematoda: Rhabditida: Strongylata (I). Beijing: Science Press; 2001.
26. Zheng, S and Fu, Z. Invertebrata, Volume 26: Granuloreticulosa: Foraminiferea, Agglutinated Foraminifera. Beijing: Science Press; 2001.
27. Gao, S, Hong, H and Zhang, S. Invertebrata, Volume 27: Cnidaria: Hydrozoa: Siphonophorae: Scyphomedusae. Beijing: Science Press; 2002.
28. Chen, Q and Shi, C. Invertebrata, Volume 28: Arthropoda: Crustacea: Amphipoda: Hyperiidea. Beijing: Science Press; 2002.
29. Dong, Z. Invertebrata, Volume 29: Mollusca: Gastropoda: Trochacea. Beijing: Science Press; 2002.
30. Chen, H and Sun, H. Invertebrata, Volume 30: Arthropoda: Crustacea: Brachyura: Marine Primitive Crabs. Beijing: Science Press; 2002.
31. Wang, Z. Invertebrata, Volume 31: Mollusca: Bivalvia: Pteriina. Beijing: Science Press; 2002.
32. Tan, Z and Su, X. Invertebrata, Volume 32: Radiolaria: Polycystinea: Nasellaria, Phaeodarea: Phaeodaria. Beijing: Science Press; 2003.
33. Sun, R and Yang, D. Invertebrata, Volume 33: Annelida: Polychaeta 2: Nereidida. Beijing: Science Press; 2004.
34. Zhang, S and Ma, X. Invertebrata, Volume 34: Mollusca: Gastropoda: Tonnacea. Beijing: Science Press; 2004.
35. Zhu, M, Song, D and Zhang, J. Invertebrata, Volume 35: Arachnida: Araneae: Tetragnathidae. Beijing: Science Press; 2003.
36. Liang, X. Invertebrata, Volume 36: Crustacea: Decapoda: Atyidae. Beijing: Science Press; 2004.
37. Chen, D and Zhang, G. Invertebrata, Volume 37: Mollusca: Gastropoda: Stylommatophora: Bradybaenidae. Beijing: Science Press; 2004.
38. Xiao, Y. Invertebrata, Volume 38: Chaetognatha: Sagittoidea. Beijing: Science Press; 2004.
39. Song, D, Zhu, M and Zhang, F. Invertebrata, Volume 39: Arachnida: Araneae: Gnaphosidae. Beijing: Science Press; 2004.
40. Liao, Y. Invertebrata, Volume 40: Echinodermata: Ophiuroidea. Beijing: Science Press; 2004.
41. Ren, X. Invertebrata, Volume 41: Crustacea: Amphipoda: Gammaridea. Beijing: Science Press; 2006.
42. Liu, R and Ren, X. Invertebrata, Volume 42: Crustacea: Cirripedia: Thoracica. Beijing: Science Press; 2007.
43. Ren, X. Invertebrata, Volume 43: Crustacea: Amphipoda: Gammaridea (II). Beijing: Science Press; 2012.
44. Li, X, Liu, R and Liang, X, et al. Invertebrata, Volume 44: Crustacea: Decapoda: Palaemonoidea. Beijing: Science Press; 2007.
45. Shen, Y and Gu, M. Invertebrata, Volume 45: Ciliophora, Oligohymenophorea, Peritrichida. Beijing: Science Press; 2016*.
46. Zhou, H, Li, F and Wang, W. Invertebrata, Volume 46: Sipuncula: Echiura. Beijing: Science Press; 2007.
47. Wu, W, Ou, J and Huang, J. Invertebrata, Volume 47: Arachnida: Acari: Phytoseiidae. Beijing: Science Press; 2009.
48. Xu, F. Invertebrata, Volume 48: Mollusca: Bivalvia: Lucinacea, Carditacea, Crassatellacea, Cardiacea. Beijing: Science Press; 2012.
49. Yang, S, Chen, H and Dai, A. Invertebrata, Volume 49: Crustacea: Decapoda: Portunidae. Beijing: Science Press; 2012.
50. Yang, T. Invertebrata, Volume 50: Tardigrada. Beijing: Science Press; 2015*.
51. Zhang, L and Fanyao, K. Invertebrata, Volume 51: Nematoda: Rhabditida: Strongylata (II). Beijing: Science Press; 2014.
52. Qiu, Z. Invertebrata, Volume 52: Platyhelminthes, Trematoda: Digenea (III). Beijing: Science Press; 2018*.
53. Sun, R and Yang, D. Invertebrata, Volume 54: Annelida: Polychaeta (III): Sabellida. Beijing: Science Press; 2014.
54. Li, F and Lin, M. Invertebrata, Volume 55: Mollusca, Gastropoda, Conidae. Beijing: Science Press; 2016*.
55. Zhang, S. Invertebrata, Volume 56: Mollusca, Gastropoda, Strombacea & Naticacea. Beijing: Science Press; 2016*.
56. Xu, F and Zhang, J. Invertebrata, Volume 57: Mollusca, Bivalvia, Tellinidae & Semelidae. Beijing: Science Press; 2017*.
57. Wu, M. Invertebrata, Volume 58: Mollusca, Gastropoda, Enoidea. Beijing: Science Press; 2018*.
58. Zhu, M, Wang, X and Zhang, Z. Invertebrata, Volume 59: Arachnida, Araneae, Agelenidae & Amaurobiidae. Beijing: Science Press; 2017*.
59. **Insects**
60. Liu, Z and W, Houyong. Insecta, Volume 1: Siphonaptera (2-Volume Set). Beijing: Science Press; 1986 (2007).
61. Chen, S. Insecta, Volume 2: Coleoptera: Hispidae. Beijing: Science Press; 1986.
62. Zhu, H and Wang, L. Insecta, Volume 3: Lepidoptera: Cyclidiidae, Drepanidae. Beijing: Science Press; 1991.
63. Xia, K. Insecta, Volume 4: Orthoptera: Acridoidea: Pamphagidae, Chrotogonid, Pyrgomorphidae. Beijing: Science Press; 1994.
64. Zhu, H and Wang, L. Insecta, Volume 5: Lepidoptera: Bombycidae, Saturniidae and Thyrididae. Beijing: Science Press; 1996.
65. Fan, Z. Insecta, Volume 6: Diptera: Calliphoridae. Beijing: Science Press; 1997.
66. Wu, C. Insecta, Volume 7: Lepidoptera, Lechithoceridae. Beijing: Science Press; 1997.
67. Lu, B. Insecta, Volume 8: Diptera: Culcidae 1. Beijing: Science Press; 1997.
68. Lu, B. Insecta, Volume 9: Diptera, Culicidae 2. Beijing: Science Press; 1997.
69. Zheng, Z and Xia, K. Insecta, Volume 10: Orthoptera: Acridoidae: Oedipodidae and Arcypteridae. Beijing: Science Press; 1998.
70. Zhu, H and Wang, L. Insecta, Volume 11: Lepidoptera: Sphingidae. Beijing: Science Press; 1997.
71. Liang, G and Zheng, Z. Insecta, Volume 12: Orthoptera: Tetrigoidea. Beijing: Science Press; 1998.
72. Ren, S. Insecta, Volume 13: Hemiptera: Heteroptera: Nabidae. Beijing: Science Press; 1998.
73. Zhang, G, Qiao, G and Zhong, T, et al. Insecta, Volume 14: Homoptera: Mindaridae and Pemphigidae. Beijing: Science Press; 1999.
74. Xue, D, Zhu, H. Insecta, Volume 15: Lepidoptera: Geometridae: Larentiinae. Beijing: Science Press; 1999.
75. Chen, Y. Insecta, Volume 16: Lepidoptera: Noctuidae. Beijing: Science Press; 1999.
76. Huang, F. Insecta, Volume 17: Isoptera. Beijing: Science Press; 2000.
77. He, J, Chen, X and Ma, Y. Insecta, Volume 18: Hymenoptera: Braconidae. Beijing: Science Press; 2000.
78. Fang, C. Insecta, Volume 19: Lepidoptera: Arctiidae. Beijing: Science Press; 2000.
79. Wu, Y. Insecta, Volume 20: Hymenoptera: Melittidae, Apidae. Beijing: Science Press; 2000.
80. Jiang, S and Chen, L. Insecta, Volume 21: Coleoptera: Cerambycidae, Lepturinae. Beijing: Science Press; 2001.
81. Wang, Z. Insecta, Volume 22: Homoptera: Coccoidea, Pseudococcidae, Aclerdidae, Eriococcidae, Asterolecaniidae, Coccidae, Cerococcidae and Lecanodiaspididae 2001.
82. Zhao, J, Liang, E and Shi, Y, et al. Insecta, Volume 23: Diptera: Tachinidae (1). Beijing: Science Press; 2001.
83. Bu, W and Zheng, L. Insecta, Volume 24: Hemiptera: Lasiochilidae, Lyctocoridae and Anthocoridae 2001.
84. Wu, C. Insecta, Volume 25: Lepidoptera: Papilionidae: Papilioninae, Zerynthiinae, Parnassiinae. Beijing: Science Press; 2001.
85. Ma, Z, Xue, W and Feng, Y. Insecta, Volume 26: Diptera: Muscidae (II): Phaoniinae (I). Beijing: Science Press; 2002.
86. Liu, Y and Li, G. Insecta, Volume 27: Lepidoptera: Tortricidae. Beijing: Science Press; 2002.
87. Yuan, F and Zhou, Y. Insecta, Volume 28: Homoptera: Membracoidea: Aetalionidae, Membracidae. Beijing: Science Press; 2002.
88. He, J and Xu, Z. Insecta, Volume 29: Hymenoptera: Dyrinidae. Beijing: Science Press; 2002.
89. Zhao, Z. Insecta, Volume 30: Lepidoptera Lymantriidae. Beijing: Science Press; 2003.
90. Wu, C and Fang, C. Insecta, Volume 31: Lepidoptera: Notodontidae. Beijing: Science Press; 2003.
91. Xia, K and Yin, X. Insecta, Volume 32: Orthoptera: Acridoidea: Gomphoceridae, Acrididae. Beijing: Science Press; 2003.
92. Zheng, L. Insecta, Volume 33: Hemiptera, Miridae, Mirinae. Beijing: Science Press; 2004.
93. Yang, D and Yang, J. Insecta, Volume 34: Diptera: Empididae: Hemerodromiinae and Hybotinae. Beijing: Science Press; 2004.
94. Chen, Y and Ma, W. Insecta, Volume 35: Dermaptera. Beijing: Science Press; 2004.
95. Zhao, Z. Insecta, Volume 36: Lepidoptera: Thyatiridae. Beijing: Science Press; 2004.
96. He, J, Chen, X and Ma, Y. Insecta, Volume 37: Hymenoptera: Braconidae II. Beijing: Science Press; 2004.
97. Zhu, H, Wang, L and Han, H. Insecta, Volume 38: Lepidoptera: Hepialidae and Epiplemidae. Beijing: Science Press; 2004.
98. Yang, J, Yang, X and Li, W. Insecta, Volume 39: Neuroptera: Chrysopidae. Beijing: Science Press; 2005.
99. Tan, J, Wang, S and Zhou, H. Insecta, Volume 40: Coleoptera: Eumolpidae: Eumolpinae. Beijing: Science Press; 2005.
100. Qiao, G and Zhong, T. Insecta, Volume 41: Homoptera: Drepanosiphidae. Beijing: Science Press; 2005.
101. Huang, D and Xiao, H. Insecta, Volume 42: Hymenoptera: Pteromalidae. Beijing: Science Press; 2005.
102. Xia, K and Li, H. Insecta, Volume 43: Orthoptera: Acridoidea: Catantopidae. Beijing: Science Press; 2006.
103. Wu, Y. Insecta, Volume 44: Hymenoptera: Megachilidae. Beijing: Science Press; 2006.
104. Ding, J. Insecta, Volume 45: Homoptera: Delphacidae. Beijing: Science Press; 2006.
105. Chen, J and Yang, J. Insecta, Volume 46: Hymenoptera: Braconidae (IV). Beijing: Science Press; 2006.
106. Wu, C and Liu, Y. Insecta, Volume 47: Lepidoptera: Lasiocampidae. Beijing: Science Press; 2006.
107. Fan, Z. Insecta, Volume 49: Diptera: Muscidae (I). Beijing: Science Press; 2008.
108. Huang, C, Cheng, X. Insecta, Volume 50: Diptera: Syrphidae. Beijing: Science Press; 2012.
109. Yang, D and Liu, X. Insecta, Volume 51: Megaloptera. Beijing: Science Press; 2010.
110. Wu, C. Insecta, Volume 52: Lepidoptera: Pieridae. Beijing: Science Press; 2010.
111. Ding, Y, Zhang, L, Wang, M, et al. Insecta, Volume 53: Diptera: Dolichopodidae (2-Volume Set). Beijing: Science Press; 2011.
112. Han, H, Xue, D. Insecta, Volume 54: Lepidoptera: Geometridae: Geometrinae. Beijing: Science Press; 2011.
113. Yuan, F, Yuan, X and Xue, G. Insecta, Volume 55: Lepidoptera: Hesperiidae. Beijing: Science Press; 2015*.
114. He, J and Xu, Z. Insecta, Volume 56: Hymenoptera: Proctotrupoidea (I) 2015*.
115. Kang, L, Liu, C and Liu, X. Insecta, Volume 57: Orthoptera: Tettigoniidae: Phaneropterinae. Beijing: Science Press; 2014.
116. Yang, D, Li, W and Zhu, F. Insecta, Volume 58: Plecoptera: Nemouroidea. Beijing: Science Press; 2015.
117. Xu, R and Sun, Y. Insecta, Volume 59: Diptera: Tabanidae. Beijing: Science Press; 2013.
118. Qiao, G, Jiang, L and Chen, J, et al. Insecta, Volume 60: Hemiptera: Hormaphididae, Phloeomyzidae. Beijing: Science Press; 2017*.
119. Yang, X, Ge, S and Wang, S, et al. Insecta, Volume 61: Coleoptera: Chrysomelidae: Chrysomelinae. Beijing: Science Press; 2014.
120. Liu, G and Zheng, L. Insecta, Volume 62: Hemiptera: Miridae (II): Orthotylinae. Beijing: Science Press; 2014.
121. Ren, G. Insecta, Volume 63: Coleoptera: Tenebrionidae (I). Beijing: Science Press; 2016*.
122. Xiao, H, Huang, D, Jiao, T. Insecta, Volume 64: Hymenoptera: Chalcidoidea: Pteromalidae (II): Pteromalinae. Beijing: Science Press; 2019*.
123. Yang, D, Dong, H and Zhang, K. Insecta, Volume 65: Diptera: Rhagionidae: Athericidae. Beijing: Science Press; 2016*.
124. Yang, M, Meng, Z and Li, Z. Insecta, Volume 67: Hemiptera: Cicadellidae (II): Cicadellinae. Beijing: Science Press; 2017*.
125. Wang, X, Zhan, Q and Wang, A. Insecta, Volume 68: Neuroptera: Myrmeleontoidea. Beijing: Science Press; 2018*.

**Table S3 List of Volumes for Florarum Cryptogamarum Sinicarum**

1. **Bryophytes**
2. Gao, Q. Flora Bryophytorum Sinicorum, Volume 1: Sphagnales, Andreaeales, Archidiales, Dicranales. Beijing: Science Press; 1994.
3. Gao, Q and Cao, T. Flora Bryophytorum Sinicorum, Volume 2: Fissidentales Pottiales. Beijing: Science Press; 1996.
4. LI, X. Flora Bryophytorum Sinicorum, Volume 3: Grimmiales, Funariales Tetraphidales. Beijing: Science Press; 2000.
5. Li, X. Flora Bryophytorum Sinicorum, Volume 4: Eubryales. Beijing: Science Press; 2006.
6. Wu, P and Jia, Y. Flora Bryophytorum Sinicorum, Volume 5: Redactores Principales. Beijing: Science Press; 2011.
7. Wu, P. Flora Bryophytorum Sinicorum, Volume 6: Hookeriales, Hypnobryales. Beijing: Science Press; 2001.
8. Hu, R and Wang, Y. Flora Bryophytorum Sinicorum, Volume 7: Hypnobryales. Beijing: Science Press; 2005.
9. Wu, P and Jia, Y. Flora Bryophytorum Sinicorum, Volume 8: Hypnobryales, Buxbaumiales, Polytrichales, Takakiales. Beijing: Science Press; 2004.
10. Gao, Q. Flora Bryophytorum Sinicorum, Volume 9: Takakiales, Calobryales Jungermanniales. Beijing: Science Press; 2003.
11. Gao, Q and Wu, Y. Flora Bryophytorum Sinicorum, Volume 10: Jungermanniales (Lophoziaceae-Neotrichocoleaceae). Beijing: Science Press; 2008.
12. **Lichens**
13. Chen, J. Flora Lichenum Sinicorum, Volume 4: Parmeliaceae(I). Beijing: Science Press; 2015*.
14. Wu, J and Liu, H. Flora Lichenum Sinicorum, Volume 11: Peltigerales (I). Beijing: Science Press; 2012.
15. Jia, Z, Wei, J. Flora Lichenum Sinicorum, Volume 13: Ostropales (I), Graphidaceae (1). Beijing: Science Press; 2016*.
16. **Fungi**
17. Zheng, R and Yu, Y. Flora Fungorum Sinicorum, Volume 1: Erysiphales. Beijing: Science Press; 1987.
18. Liu, B. Flora Fungorum Sinicorum, Volume 2: Tremellales at Dacrymycetales. Beijing: Science Press; 1992.
19. Zhao, J. Flora Fungorum Sinicorum, Volume 3: Polyporaceae. Beijing: Science Press; 1998.
20. Hu, Y. Flora Fungorum Sinicorum, Volume 4: Meliolales (I). Beijing: Science Press; 1996.
21. Qi, Z. Flora Fungorum Sinicorum, Volume 5: Aspergillus et Teleomorphi Cognati. Beijing: Science Press; 1997.
22. Yu, Y. Flora Fungorum Sinicorum, Volume 6: Peronosporales. Beijing: Science Press; 1998.
23. Liu, B. Flora Fungorum Sinicorum, Volume 7: Hymenogastrales, Melanogastrales. Beijing: Science Press; 1998.
24. Zhuang, W. Flora Fungorum Sinicarum, Volume 8: Sclerotiniaceae et Geoglossaceae. Beijing: Science Press; 1998.
25. Liu, X and Guo, Y. Flora Fungorum Sinicorum, Volume 9: Pseudocercospora. Beijing: Science Press; 1998.
26. Wang, Y. Flora Fungorum Sinicorum, Volume 10: Meliolales (I). Beijing: Science Press; 1998.
27. Hu, Y. Flora Fungorum Sinicorum, Volume 11: Meliolales (II). Beijing: Science Press; 1998.
28. Guo, L. Flora Fungorum Sinicorum, Volume 12: Ustilaginaceae. Beijing: Science Press; 2000.
29. Li, Z. Flora Fungorum Sinicorum, Volume 13: Entomophtorales. Beijing: Science Press; 2000.
30. Zhang, Z. Flora Fungorum Sinicorum, Volume 14: Cladosporium, Fusicladium, Pyricularia. Beijing: Science Press; 2003.
31. Bai, J. Flora Fungorum Sinicorum, Volume 15: Sphaeropsidales: Phoma Phyllosticta: Redactores principales. Beijing: Science Press; 2003.
32. Zhang, T. Flora Fungorum Sinicorum, Volume 16: Alternaria. Beijing: Science Press; 2003.
33. Bai, J. Flora Fungorum Sinicorum, Volume 17: Sphaeropsidales: Ascochyta Septoria: Redactor Principalis. Beijing: Science Press; 2003.
34. Zhang, X and Zhao, J. Flora Fungorum Sinicorum, Volume 18: Ganodermataceae. Beijing: Science Press; 2000.
35. Zhuang, J. Flora Fungorum Sinicorum, Volume 19: Uredinales (II). Beijing: Science Press; 2003.
36. Guo, L and Liu, X. Flora Fungorum Sinicorum, Volume 20: Mycovellosiella Passalora Phaeoramularia. Beijing: Science Press; 2003.
37. Zhuang, W. Flora Fungorum Sinicorum, Volume 21: Hyaloscyphaceae, Sarcoscyphaceae et Sarcosomataceae. Beijing: Science Press; 2004.
38. Zang, M. Flora Fungorum Sinicorum, Volume 22: Boletaceae (I). Beijing: Science Press; 2006.
39. Liu, B. Flora Fungorum Sinicorum, Volume 23: Schlerodermatales Tulostomatales Phallales et Podaxales. Beijing: Science Press; 2005.
40. Guo, Y and Liu, X. Flora Fungorum Sinicorum, Volume 24: Cercospora. Beijing: Science Press; 2005.
41. Zhuang, J. Flora Fungorum Sinicorum, Volume 25: Uredinales (III). Beijing: Science Press; 2005.
42. Zhang, Z. Flora Fungorum Sinicorum, Volume 26: Botrytis Ramularia. Beijing: Science Press; 2006.
43. Yang, Z. Flora Fungorum Sinicorum, Volume 27: Amanitaceae. Beijing: Science Press; 2005.
44. Shen, Y and Ye, D. Flora Fungorum Sinicorum, Volume 28: Laboulbeniales. Beijing: Science Press; 2006.
45. Zhang, X and Dai, Y. Flora Fungorum Sinicorum, Volume 29: Hymenochaetaceae. Beijing: Science Press; 2005.
46. Zhang, T. Flora Fungorum Sinicorum, Volume 30: Helminthosporioid Hyphomycetes. Beijing: Science Press; 2010.
47. Zhang, T. Flora Fungorum Sinicorum, Volume 31: 26 Genera of Dematiaceous Dictyosporous Hyphomycetes excluding Alternaria. Beijing: Science Press; 2009.
48. Liang, Z. Flora Fungorum Sinicorum, Volume 32: Cordyceps. Beijing: Science Press; 2007.
49. Zhang, K, Mo, M. Flora Fungorum Sinicorum, Volume 33: Arthrobotrys et Genera Cetera Cognata. Beijing: Science Press; 2006.
50. Qi, P, Jiang, Z and Xiang, M. Flora Fungorum Sinicorum, Volume 34: Phomopsis. Beijing: Science Press; 2007.
51. Kong, H. Flora Fungorum Sinicorum, Volume 35: Penicillium et Teleomorphi Cognati Redactor Principalis. Beijing: Science Press; 2007.
52. Zhou, T, Chen, Y and Zhao, L, et al. Flora Fungorum Sinicorum, Volume 36: Geastraceae Nidulariceae. Beijing: Science Press; 2007.
53. Wu, W. Flora Fungorum Sinicorum, Volume 37: Sporidesmium et Genera Cognatas. Beijing: Science Press; 2009.
54. Ge, Q, Chen, Y and Xu, T. Flora Fungorum Sinicorum, Volume 38: Pestalotiopsis. Beijing: Science Press; 2009.
55. Guo, L. Flora Fungorum Sinicorum, Volume 39: Tilletiales, Urocystidales, Entorrhizales, Doassansiales, Entylomatales, and Georgefischeriales. Beijing: Science Press; 2011.
56. Lin, Y. Flora Fungorum Sinicorum, Volume 40: Rhytismatales. Beijing: Science Press; 2012.
57. Zhuang, J. Flora Fungorum Sinicorum, Volume 41: Uredinales (4). Beijing: Science Press; 2012.
58. Dai, Y and Xiong, H. Flora Fungorum Sinicorum, Volume 42: Corticiaceae s.l. (I). Beijing: Science Press; 2012.
59. Liang, Z. Flora Fungorum Sinicorum, Volume 43: Paecilomyces, Isaria and Taifangflania. Beijing: Science Press; 2013.
60. Zang, M. Flora Fungorum Sinicorum, Volume 44: Boletaceae (II). Beijing: Science Press; 2013.
61. Li, Y and Tolgor, B. Flora Fungorum Sinicorum, Volume 45: Pleurotoid-Lentinoid Fungi. Beijing: Science Press; 2014.
62. Zhang, Z and Zhang, T. Flora Fungorum Sinicorum, Volume 46: Phyllachora. Beijing: Science Press; 2014.
63. Zhuang, W. Flora Fungorum Sinicorum, Volume 47: Nectriaceae et Bionectriaceae. Beijing: Science Press; 2013.
64. Zhuang, W. Flora Fungorum Sinicorum, Volume 48: Pyronemataceae. Beijing: Science Press; 2014.
65. Tolgor, B. Flora Fungorum Sinicorum, Volume 49: Strophariaceae (1). Beijing: Science Press; 2013.
66. Guo, L, Li, Z and Lu, C, et al. Flora Fungorum Sinicorum, Volume 50: Exobasidiales, Septobasidiales. Beijing: Science Press; 2015*.
67. Zhang, T. Flora Fungorum Sinicorum, Volume 51: Dematiaceous Hyphomycetes from Soil. Beijing: Science Press; 2019*.
68. Yang, Z. Flora Fungorum Sinicorum, Volume 52: Fungi Lepiotoidei: Agaricaceae. Beijing: Science Press; 2019*.
69. Fan, L. Flora Fungorum Sinicorum, Volume 54: Lycoperdales: Lycoperdaceae, Mycenastraceae. Beijing: Science Press; 2019*.
70. Zhang, X. Flora Fungorum Sinicorum, Volume 55: Corynespora et Genera Cognata. Beijing: Science Press; 2018*.
71. Zhuang, W. Flora Fungorum Sinicorum, Volume 56: Helotiaceae Redactor Principalis. Beijing: Science Press; 2018*.
72. Dai, Y. Flora Fungorum Sinicorum, Volume 57: Hymenochaetales (1) Redactor Principalis. Beijing: Science Press; 2018*.
73. Guo, L. Flora Fungorum Sinicorum, Volume 59: Xylaria. Beijing: Science Press; 2019*.
74. Li, Y. Flora Fungorum Sinicorum, Myxomycetes I: Ceratiomyxales, Echinosteliales, Liceales, Trichiales. Beijing: Science Press; 2008.
75. Li, Y. Flora Fungorum Sinicorum, Myxomycetes II: Physarales and Stemonitales. Beijing: Science Press; 2008.
76. **Algae**
77. Xia, B. Flora Algarum Marinarum Sinicarum, Tomus 1: Cyanophyta. Beijing: Science Press; 2017*.
78. Zheng, B and Li, J. Flora Algarum Marinarum Sinicarum, Tomus 2: Rhodophyta (No. 1) Porphyridiales, Erythropeltidales, Goniotrichales, Bangiales. Beijing: Science Press; 2009.
79. Zeng, C. Flora Algarum Marinarum Sinicarum, Tomus 2: Rhodophyta (No. 2) Acrochetiales, Nemaliales, Bonnemaisoniales. Beijing: Science Press; 2005.
80. Xia, B. Flora Algarum Marinarum Sinicarum, Tomus 2: Rhodophyta (No. 3) Gelidiales, Cryptonemiales, Hildenbrandiales. Beijing: Science Press; 2004.
81. Xia, B, Wang, Y and Zhou, J, et al. Flora Algarum Marinarum Sinicarum, Tomus 2: Rhodophyta (No. 4) Corallinales. Beijing: Science Press; 2013.
82. Zhang, J and Xia, B. Flora Algarum Marinarum Sinicarum, Tomus 2: Rhodophyta (No. 5) Ahnfeltiales, Gigartinales, Sebdeniales, Nemastomatales, Plocamiales, Gracilariales, Rhodymeniales. Beijing: Science Press; 1999.
83. Zheng, B. Flora Algarum Marinarum Sinicarum, Tomus 2: Rhodophyta (No. 6) Ceramiales (1): Ceramiaceae, Dasyaceae, Delesseriaceae. Beijing: Science Press; 2001.
84. Xia, B. Flora Algarum Marinarum Sinicarum, Tomus 2: Rhodophyta (No. 7) Ceramiales: Rhodomelaceae. Beijing: Science Press; 2011.
85. Luan, R. Flora Algarum Marinarum Sinicarum, Tomus 3: Phaeophyta (No. 1). Beijing: Science Press; 2013.
86. Zeng, C and Lu, B. Flora Algarum Marinarum Sinicarum, Tomus 3: Phaeophyta (No. 2) Fucales. Beijing: Science Press; 2000.
87. Ding, L. Flora Algarum Marinarum Sinicarum, Tomus 4: Chlorophyta (No. 1) Ulotrichales, Chaetophorales, Phaeophilales, Ulvales, Prasiolales, Cladophorales, Acrosiphoniales. Beijing: Science Press; 2013.
88. Guo, Y. Flora Algarum Marinarum Sinicarum, Tomus 5: Bacillariophyta (No. 1) Centricae. Beijing: Science Press; 2003.
89. Cheng, Z, Gao, Y and Liu, S, et al. Flora Algarum Marinarum Sinicarum, Tomus 5: Bacillariophyta (No. 2) Pennatae I. Beijing: Science Press; 2012.
90. Cheng, Z and Gao, Y. Flora Algarum Marinarum Sinicarum, Tomus 5: Bacillariophyta (No. 3) Pennatae (2) Naviculales: Naviculaceae, Cymbellaceae, Auriculaceae, Gomphonemaceae. Beijing: Science Press; 2013.
91. Lin, Y. Flora Algarum Marinarum Sinicarum, Tomus 6: Pyrrophyta (No. 1) Dinophyceae , Ceratiaceae. Beijing: Science Press; 2009.
92. Rao, Q. Flora Algarum Sinicarum Aquae Dulcis, Volume 1: Zygnemataceae. Beijing: Science Press; 1988.
93. Zhu, H. Flora Algarum Sinicarum Aquae Dulcis, Volume 2: Chroococcophyceae. Beijing: Science Press; 1991.
94. Han, F. Flora Algarum Sinicarum Aquae Dulcis, Volume 3: Charophyta. Beijing: Science Press; 1994.
95. Qi, Y. Flora Algarum Sinicarum Aquae Dulcis, Volume 4: Bacillariophyta: Centricae. Beijing: Science Press; 1995.
96. Li, S and Bi, L. Flora Algarum Sinicarum Aquae Dulcis, Volume 5: Ulothricales, Ulvalves, Chaetophorales, Trentepholiales, Sphaeropleales. Beijing: Science Press; 1998.
97. Shi, Z. Flora Algarum Sinicarum Aquae Dulcis, Volume 6: Euglenophyta. Beijing: Science Press; 1999.
98. Wei, Y. Flora Algarum Sinicarum Aquae Dulcis, Volume 7: Chlorophyta: Zygnnmatales, Mesotaeniaceae, Desmidiales, Desmidiaceae (1). Beijing: Science Press; 2003.
99. Bi, L and Hu, Z. Flora Algarum Sinicarum Aquae Dulcis, Volume 8: Chlorophyta: Chlorococcales (1). Beijing: Science Press; 2005.
100. Zhu, H. Flora Algarum Sinicarum Aquae Dulcis, Volume 9: Cyanophyta: Hormogonophyceae. Beijing: Science Press; 2007.
101. Qi, Y and Li, J. Flora Algarum Sinicarum Aquae Dulcis, Volume 10: Bacillariophyta: Pennatae. Beijing: Science Press; 2004.
102. Wang, Q. Flora Algarum Sinicarum Aquae Dulcis, Volume 11: Xanthophyta. Beijing: Science Press; 2007.
103. Shi, Z. Flora Algarum Sinicarum Aquae Dulcis, Volume 12: Bacillariophyta: Gomphonemaceae. Beijing: Science Press; 2005.
104. Shi, Z. Flora Algarum Sinicarum Aquae Dulcis, Volume 13: Rhodophyta: Phaeophyta. Beijing: Science Press; 2006.
105. Li, J and Qi, Y. Flora Algarum Sinicarum Aquae Dulcis, Volume 14: Bacillariophyta: Naviculaceae (I). Beijing: Science Press; 2010.
106. Liu, G and Hu, Z. Flora Algarum Sinicarum Aquae Dulcis, Volume 15: Chlorophyta: Chlorococcales (II) Tetrasporales Dichotomosiphonales Cladophorales. Beijing: Science Press; 2012.
107. Shi, Z. Flora Algarum Sinicarum Aquae Dulcis, Volume 16: Bacillariophyta: Cymbellaceae. Beijing: Science Press; 2013.
108. Wei, Y. Flora Algarum Sinicarum Aquae Dulcis, Volume 17: Chlorophyta: Desmidiales Desmidiaceae, Sectio II: Actinotaenium, Cosmarium, Cosmocladium. Beijing: Science Press; 2013.
109. Wei, Y. Flora Algarum Sinicarum Aquae Dulcis, Volume 18: Chlorophyta: Desmidiales Desmidiaceae Sectio III Xanthidium, Staurodesmus, Staurastrum, Filamentous Desmids. Beijing: Science Press; 2014.
110. Li, J and Qi, Y. Flora Algarum Sinicarum Aquae Dulcis, Volume 19: Bacillariophyta: Naviculaceae (II). Beijing: Science Press; 2014.

Hu, H. Flora Algarum Sinicarum Aquae Dulcis, Volume 20: Chlorophyta: Chlorophyceae: Order Volvocales (II) Genus Chlamydomonas. Beijing: Science Press; 2015

Figure S1 Representative floristic publications in China


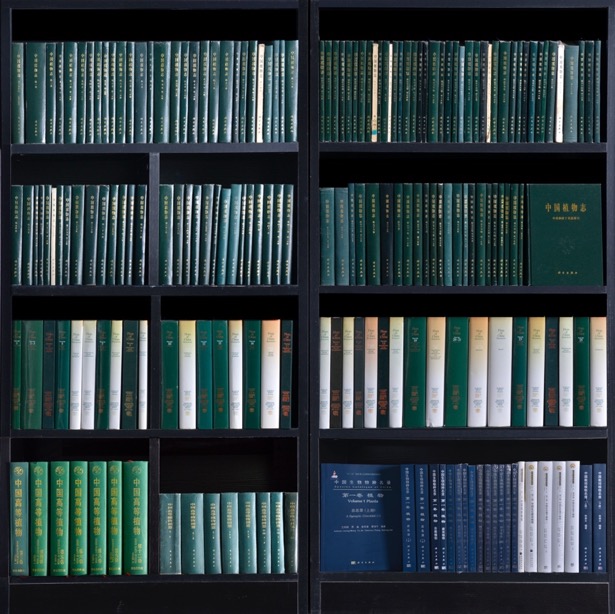


Publications on the flora in China including *Flora Reipublicae Popularis Sinicae* on the top two shelves, *Flora of China* on the third shelf, *Higher Plants of China* and *Illustrated Flora of Higher Plants in China* on the left of the bottom shelf and *Species Catalogue of China (Higher Plants)*, *Fossil Flora of China* and *A Catalogue of Exotic and Cultivated Plants of China* on the right of the bottom shelf.
